# Marine Natural Products in Clinical Use

**DOI:** 10.3390/md20080528

**Published:** 2022-08-18

**Authors:** Neshatul Haque, Sana Parveen, Tingting Tang, Jiaen Wei, Zunnan Huang

**Affiliations:** 1 Key Laboratory of Big Data Mining and Precision Drug Design of Guangdong Medical University, Key Laboratory of Computer-Aided Drug Design of Dongguan City, Key Laboratory for Research and Development of Natural Drugs of Guangdong Province, School of Pharmacy, Guangdong Medical University, No. 1 Xincheng Road, Dongguan 523808, China; neshathaq@gmail.com (N.H.); tingtingtang0430@163.com (T.T.); jiaenwei8@163.com (J.W.); 2 Centre for Cellular & Molecular Biology (CCMB), Hyderabad 500007, India; sana26jan@gmail.com; 3 Southern Marine Science and Engineering Guangdong Laboratory, Zhanjiang 524023, China

**Keywords:** marine natural products, marine drugs, spongonucleosides, microtubule inhibitors, DNA alkylating agent, drug conjugated with an antibody, peptides or proteins, fish oil

## Abstract

Marine natural products are potent and promising sources of drugs among other natural products of plant, animal, and microbial origin. To date, 20 drugs from marine sources are in clinical use. Most approved marine compounds are antineoplastic, but some are also used for chronic neuropathic pain, for heparin overdosage, as haptens and vaccine carriers, and for omega-3 fatty-acid supplementation in the diet. Marine drugs have diverse structural characteristics and mechanisms of action. A considerable increase in the number of marine drugs approved for clinical use has occurred in the past few decades, which may be attributed to increasing research on marine compounds in laboratories across the world. In the present manuscript, we comprehensively studied all marine drugs that have been successfully used in the clinic. Researchers and clinicians are hopeful to discover many more drugs, as a large number of marine natural compounds are being investigated in preclinical and clinical studies.

## 1. Introduction

A large portion of Earth, approximately two-thirds, is water, which holds enormous amounts of treasures, such as foods and medicines. Human civilization has been consuming sea products such as sea salt as a food ingredient, which is obtained by direct evaporation of sea water, and other sea food throughout history. With the discovery of cytotoxic compound arabinosyl thymidine (spongothymidine) in the 1950s [1,2], the interest and hope of various chemists and biomedical researchers peaked for the investigation of natural products of marine origin for therapeutic benefits. Following the first discovery, numerous marine compounds or synthetic analogs of marine compounds were discovered by dedicated scientists and researchers around the world [3,4]. Before 1970, two therapeutically important compounds of marine origin, cytarabine (arabinosyl cytosine, ara-C) and protamine sulfate, were in clinical use, followed by vidarabine (arabinosyl adenine, ara-A) in 1976. After nearly three decades, another marine compound, ziconotide, was approved for clinical use. Since then, researchers have expressed considerable interest in investigating marine life for drug discovery.

In this review, we present drugs from marine compounds or compounds inspired by marine natural products that are approved for clinical use and are also available on the market. The purpose of this review is to provide readers with information on the importance of marine products in human health, which is anticipated to solve broader areas of disease-related complications in the future. This review is not limited to information on marine drugs; it also includes their structural aspects and mechanisms of action involved in alleviating pathophysiological conditions. We hope to draw the attention of researchers who are working in the drug design field, especially those inspired by marine natural resources.

## 2. Marine Bioactive Compounds Available on the Market

A brief overview of the marine drugs discussed in this review is presented in Table 1 in the most relevant manner to follow the topic-wise discussion of marine drugs. Table 1 also provides an understanding of the diversity of marine drugs in clinical use. These drugs are classified into six categories, where the basis of classification is nonuniform but maintains the flow of context within the category. Most of the drugs are categorized on the basis of the complexity of structures such as “spongonucleosides”, “antibody-drug conjugates”, and “peptides or proteins used as drugs or used in drug preparations”, but some are categorized on the basis of their mechanism of action, such as “microtubule inhibitors” and “deoxyribonucleic acid (DNA) alkylating agents”, or their natural source of abundance, such as “fish oil and its components as drugs”. Table 1 briefly summarizes important and relevant information along with the indications for clinical use and the mechanisms of action of drugs.

### 2.1. Nucleoside Analogs

**Figure 1:**
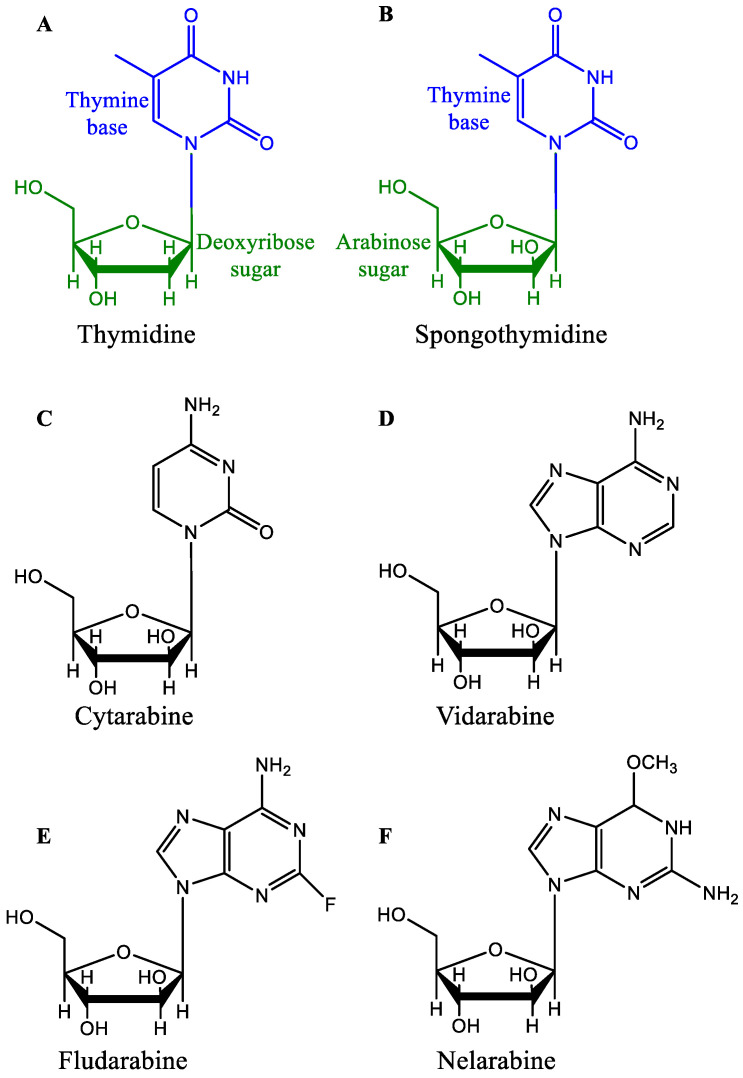
The structures of thymidine and other arabinosides.

A synthetic analog of arabinose nucleoside contains arabinose as a sugar in place of the ribose sugar and acts as an antimetabolite by competing for enzyme systems and interfering with natural biological processes of DNA or ribonucleic acid (RNA) synthesis. According to a literature review conducted by the British Medical Journal, antimetabolites that block nucleic-acid synthesis are usually studied under three categories: folic acid antagonists, purine antagonists, and pyrimidine antagonists. Synthetic analogs of arabinose nucleoside are either purine antagonists (vidarabine, fludarabine, and nelarabine) or pyrimidine antagonists (cytarabine) [33]. Arabinose nucleoside synthetic analogs may be incorporated into nucleic acids or inhibit nucleic acid biosynthesis enzymes, resulting in their antiviral and antitumor properties [34,35] (Figure 1).

The first natural nucleosides known from marine ecosystems were thymidine- and uridine-like molecules extracted from the marine sponge *Cryptotethia crypta* [1,2]. Due to their physicochemical properties and nature of origin, these molecules were named spongothymidine (ara-T) and spongouridine (ara-U), collectively called spongonucleosides [1,2]. Soon after the discovery of spongonucleosides, chemists began to synthesize the arabinose nucleosides ara-A [36,37], ara-G [38,39], ara-C [40], and ara-U [41], which were later also extracted from natural marine sources such *Eunicella cavolini* (yellow sea whip) [42].

#### 2.1.1. Cytarabine

**Figure 2:**
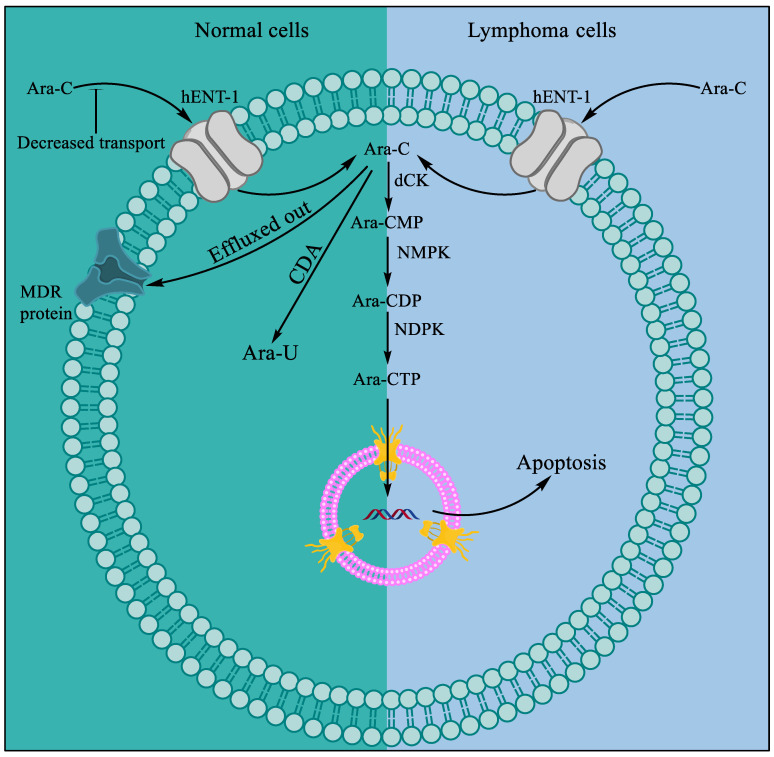
Mechanism of resistance to ara-C in normal cells and cytotoxicity in lymphoma. Compared to normal cells, lymphoma cells show more influx and less efflux of ara-C. A series of phosphorylation events by deoxycytidine kinase (dCK), followed by nucleoside monophosphate kinase (NMPK) and nucleoside diphosphate kinase (NDPK), occurs that convert ara-C to bioactive ara-CTP.

Cytarabine, aka 1-β-d-Arabinofuranosylcytosine, Ara-C, CYTOSAR-U^®^ (Pfizer, New York City, NY, USA), and DEPOCYT^®^ (Pacira Pharma, San Diego, CA, USA; Bedford Lab, Seattle, DC, USA, Enzon Pharmaceuticals, Piscataway, NJ, USA), (Figure 1C) is the synthetic analog of naturally occurring spongothymidine. Cytarabine is among the most successful antitumor agents and is used even today, more than 50 years after its first approval in 1969 [5,43], for the treatment of acute leukemia [44]. Cytarabine is a prodrug and is phosphorylated intracellularly to produce the active molecule ara-cytidine-5′-triphosphate (ara-CTP). Intracellular conversion of ara-C to ara-CTP increases the bioactive concentration of ara-CTP before it acts rapidly as a potent inhibitor of mammalian DNA synthesis (Figure 2) [45,46,47,48]. The cytotoxic effect is mediated by inhibiting DNA polymerase, leading to megaloblastosis [46], as well as by the incorporation of ara-CTP in the growing DNA, resulting in a faulty DNA strand [34,35]. The faulty DNA eventually matures into aberrant chromosomes with multiple chromatid breaks and fragmentation in S phase of the cell cycle, which leads to cell death [49,50].

Ara-C is readily deaminated to its inactive form, uracil arabinoside, by cytidine deaminase (CDA) present in the intestine and liver, when administered orally [35,51]. Intravenous administration of ara-C produces comparatively higher active drug concentrations in plasma and cerebrospinal fluid, where 80% of the drug is excreted as uracil arabinoside in urine [52]. In 1999, DepoCyt^®^ (Pacira Pharmaceuticals, San Diego, CA, USA), a liposomal formulation of cytarabine for sustained delivery of the drug, was approved by the US Food and Drug Administration (FDA) for lymphomatous meningitis treatment [6,53]. The liposomal formulation of cytarabine alone or in combination with anthracyclines such as daunorubicin is more effective because of slow and sustained delivery and possible evasion of nanosized liposomes from the drug efflux pump as they enter the cells intact [54,55,56]. The common adverse events observed in patients receiving cytarabine treatment include anorexia, nausea, vomiting, diarrhea, bleeding, and myelosuppression. Following prolonged intravenous or intrathecal administration of cytarabine, risks for central nervous system (CNS) toxicity and renal and hepatic failure have been reported [57,58].

An intriguing question about the specificity of cytarabine toward leukemia with respect to other carcinomas has also been investigated since its discovery. Some leukemias often show acquired resistance to cytarabine, and many carcinomas show intrinsic resistance to cytarabine, which may be attributed to differences in the nucleoside transport mechanisms and the catalysis of nucleosides compared to sensitive hematological carcinomas. Researchers have hypothesized and often substantiated that, in resistant leukemic cell lines, cytarabine-nonresponding patients, or patients with other carcinomas, multiple factors, such as decreased transport of the drug in the cells by human equilibrative nucleoside transporters (hENT1–hENT4), higher efflux activity by multidrug-resistant (MDR) gene products, greater deamination of the drug to inactive uridine arabinoside by cytidine deaminase, and reduced phosphorylation by deoxycytidine kinase (dCK), may be responsible for the development of drug resistance (Table 1 and Figure 2) [57,58,59,60,61,62,63,64]. Knowledge of the resistance mechanism might be helpful in designing new therapeutic combinations, such as combination drugs and gene therapy, and for the exploration of broad-spectrum use of cytotoxic agents, such as cytarabine, in cancer treatment [62,63].

#### 2.1.2. Vidarabine

Vidarabine, aka 9-β-D-Arabinofuranosyladenine, Ara-A, and VIRA-A^®^ (King Pharmaceuticals, Bristol, TN, USA), (Figure 1D) is a synthetic analog of arabinonucleosides inspired by naturally occurring spongothymidine and spongouridine. Vidarabine selectively inhibits DNA viruses [65,66] but exhibits little or no inhibition of RNA viruses [67]. Vidarabine was principally synthesized as a potential anticancer drug [36] but was formally approved by the US FDA in 1976 as an ophthalmic ointment (3%) with indications for the treatment of superficial keratosis resistant to idoxuridine caused by herpes simplex virus (HSV), keratoconjunctivitis, and recurrent epithelial keratitis caused by HSV1/2 (Table 1) [7]. However, the drug was discontinued from the market because of the availability of a better commercial alternative [4,68]. The therapeutically active concentration of the drug was not reached because of its relatively low solubility and high body clearance, and the drug is rapidly deaminated to its inactive form arabinosyl hypoxanthine by adenosine deaminase, restricting its clinical use to limited pathological conditions [48,69]. However, the drug was investigated on multiple occasions in combination with the adenosine deaminase inhibitor deoxycoformycin (dCF, pentostatin) but had little success [70,71]. Soon, attention was switched to the more potent alternative fludarabine (discussed in the next section) [36,48,72].

#### 2.1.3. Fludarabine

Fludarabine, aka 9-β-d-Arabinofuranosyl-2-fluoroadenine-5’-phosphate, FLUDARA^®^ (Sandoz, Basel, Switzerland) or OFORTA^®^ (Sanofi-Aventis, Paris, France), (Figure 1E) is a synthetic purine nucleoside analog inspired by marine spongothymidine. The drug was initially approved for the treatment of adult B-cell chronic lymphocytic leukemia (CLL) in 1991 (Table 1) [8]. Fludarabine is more effective as a single agent for the treatment of CLL [8,73] than traditional chemotherapy using alkylating agents such as chlorambucil [74]. However, the administration of fludarabine in combination with other agents, such as cyclophosphamide, rituximab, mitoxantrone, and dexamethasone, has been proven to be a more effective treatment for CLL [75,76]. Fludarabine failed to produce therapeutic benefits in patients with other solid tumors because of the dose-limiting toxicity associated with myelosuppression [77,78,79,80]. Although fludarabine has a higher response rate than previous combination chemotherapeutic regimens, patients under treatment develop moderate to severe toxicity [8]. Severe indications of (a) bone marrow toxicity, such as thrombocytopenia and leukopenia [81,82], (b) pulmonary toxicity, such as cough, dyspnea, and hypoxia [83,84,85], and (c) neurotoxicity, such as visual field deficits, seizures, and encephalopathy [86,87], should be closely monitored in patients treated with a high dose of fludarabine to alleviate or mitigate the symptoms [8]. The mechanism of neurotoxicity remains elusive, but the evidence supports that, like adenosine, fludarabine crosses the blood-brain barrier and acts as an A1 receptor agonist, causing abnormal synaptic function [88,89]. On the other hand, pulmonary toxicity usually responds to steroids [8,90].

Fludarabine, a phosphorylated form of fluorinated arabinofuranosyl adenine, is resistant to deamination and more soluble than ara-A [91]. The drug is metabolized to a dephosphorylated form (F-ara-A) before being transported into the cell. The drug F-ara-A is administered orally, as well as parentally [48], and the clearance is mostly through urine, the degree of which depends on the dosage amount, duration, schedule, and patient metabolism [48,92,93]. F-ara-A is rephosphorylated to F-ara-adenosine-triphosphate (F-ara-ATP) by subsequent phosphorylation in the cell [94,95], where it accumulates to the bioactive concentration required for cytotoxicity by competing with cellular nucleotides and incorporating into the nucleic acid [96,97,98].

#### 2.1.4. Nelarabine

Nelarabine, aka 2-Amino-9-α-d-arabinofuranosyl-6-methoxy-9*H*-purine, ARRANON^®^ (GSK, Brentford, UK) ATRIANCE^®^ (Novartis, Basel, Switzerland), (Figure 1F) is also a synthetic purine nucleoside analog inspired by marine spongothymidine. Nelarabine is a chemotherapeutic anticancer prodrug that is first metabolized in cells to ara-G followed by phosphorylation to the active form ara-guanosine triphosphate (ara-GTP). Ara-GTP competes with dGTP for DNA polymerase and is incorporated into the nucleic acid, causing DNA breakage and cell death [99,100]. Unlike the previously mentioned nucleoside analogs, nelarabine is more water-soluble [99,100]. The half-life of the drug is 30 min, and it is mostly excreted out with urine. Inspired by the cytotoxic properties of cytarabine and fludarabine, nelarabine was tested against various hematological malignancies and was approved by the FDA in 2005 (Table 1) [9] due to its selective cytotoxic activity against T-cell acute lymphoblastic leukemia (ALL) or T-cell lymphoblastic lymphoma (ABL). Another rationale for investigating ara-G analogs as treatments for T-cell malignancies is based on the report that patients with purine nucleoside phosphorylase deficiency, an autosomal-recessive rare disease, suffer from T-cell lymphopenia-related immunodeficiency. This observation led to the hypothesis that the water-soluble purine analog might exert selective cytotoxic effects on T-cell-related malignancies [9,101,102]. The most likely cause of the T-cell toxicity of nelarabine is the higher concentration of ara-G in T cells than in B cells [101,102].

Nonhematological adverse events associated with nelarabine treatment are mostly mild to moderate and are either reversible or can be alleviated by the appropriate medications. Neurological adverse events that may be severe mainly limit the use of nelarabine for ALL and ABL. Patients administered nelarabine treatment must be kept under close observation, and the treatment should be stopped at any sign of neurological adverse events [102,103,104].

#### 2.1.5. Histochrome^®^: Sodium Salt of Echinochrome A—A Common Sea Urchin Pigment

**Figure 3:**
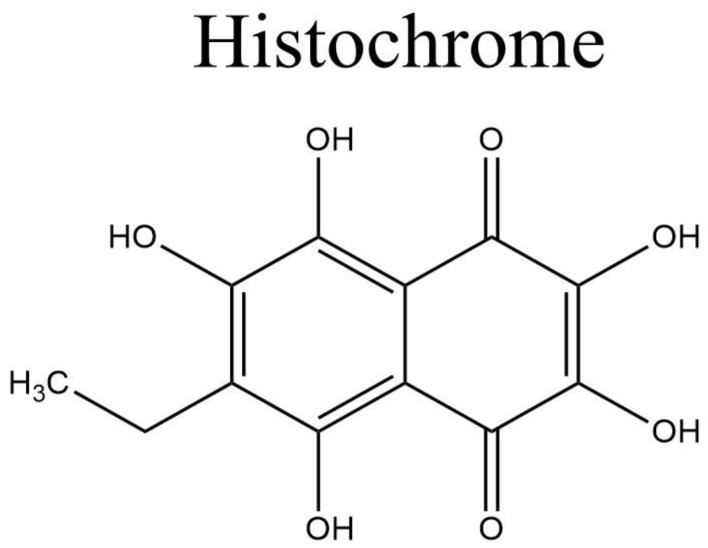
Histochrome structure.

Histochrome^®^ from Pacific-Ocean Institute of Bioorganic Chemistry (Vladivostok, Russia) (Figure 3) is another secondary metabolite from the marine organism *Scaphechinus mirabilis* (sea urchin), which has been in clinical use since 1999 [10,105]. The drug exerts a therapeutic cytoprotective effect and is used mostly in Russia to treat a variety of diseases, such as degeneration of the macula, retina, and cornea, circulatory disorder of the retina, and myocardial ischemia/reperfusion injury [106]. The cytoprotective action of the drug is derived from its ability to protect against DNA damage by regulating the apoptosis cascade [105].

### 2.2. Microtubule Inhibitors

**Figure 4:**
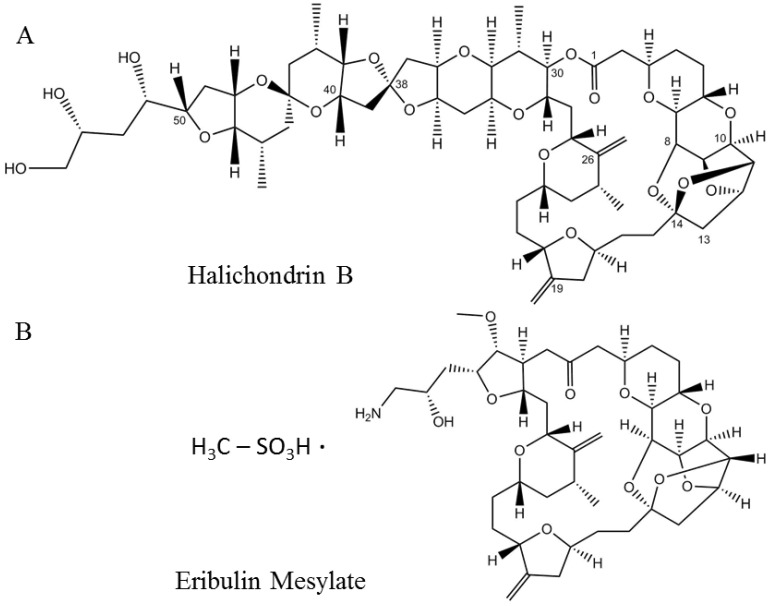
Structures of the naturally occurring marine compound (**A**) halichondrin B and its synthetic analog (**B**) eribulin mesylate.

Eribulin mesylate or EM, aka E7389 and HALAVEN^®^ (Eisai, Bunkyo, Japan) (Figure 4B) is a synthetic analog of halichondrin B (Figure 4A), a polyether macrolide discovered in the marine sponge *Halichondria okadai* [107]. Similar to halichondrin B, EM also inhibits tumor cell proliferation by arresting cells in G2–M phase of the cell cycle [107,108,109]. EM was approved as a treatment for metastatic breast cancer in patients with a previous history of chemotherapeutic treatment in 2010 by the US FDA (Table 1) [110] and in 2011 by Health Canada [11]. In the following year (2016), EM was also approved for the treatment of metastatic liposarcoma (Table 1) [12,109,111].

Halichondrin B belongs to the family of halichondrin natural compounds. Members of this chemical class are categorized into halichondrin, norhalichondrin, and homohalichondrin [107]. Halichondrin B is a more potent inhibitor than most of the known microtubule inhibitors and works by producing nonproductive aggregates of tubulin [108]. The rare abundance of the organism and tedious process of the total synthesis of such a large polyether macrolide (1111.329 Da) encouraged the discovery of a minimum pharmacophore analog by the Kishi group in 2001 [112], which was named ER-086526 (later renamed eribulin, E7389). EM was shown to inhibit the proliferation of multiple cell lines at subnanomolar concentrations through a mechanism consistent with the parent molecule halichondrin B [112].

With the promising results from phase I and phase II trials of EM, a phase III trial was conducted with 762 patients with breast cancer who underwent numerous treatments, between two and five previous chemotherapy rounds, with anthracycline and a taxane [113]. The patients were treated either with eribulin or the treatment of physician’s choice (TPC), including treatment with any single agent such as chemotherapy or hormonal or biological therapy approved for the disease. The improvement in overall survival (OS) in women treated with eribulin (13.1 months) was clinically meaningful compared with TPC (10.6 months). Eribulin remains unmetabolized and is excreted unchanged in feces with a half-life of 40 h [114]. Manageable adverse events were common in patients receiving both treatments, except for peripheral neuropathy observed in the eribulin treatment group, which resulted in the discontinuation of treatment in 5% (of 503 patients) of the patients [115,116]. EM was initially approved for patients with HER2-negative metastatic breast cancer, but it was also shown to lead to an effective clinical outcome for HER2-positive patients, indicating a broader use of the drug in the future [111,117].

### 2.3. DNA Alkyating Agents

DNA alkylating agents attach alkyl groups to multiple guanine nucleobases, rendering them unable to function properly in dividing cells, leading to cell death. Alkylating agents are sometimes specific for minor or major grooves of a sequence and can alkylate a single guanine base or bridge two guanine bases of the same or different DNA strands of the same DNA [118]. Trabectedin and lurbinectedin are two popular DNA alkylating agents from marine sources.

#### 2.3.1. Trabectedin

**Figure 5:**
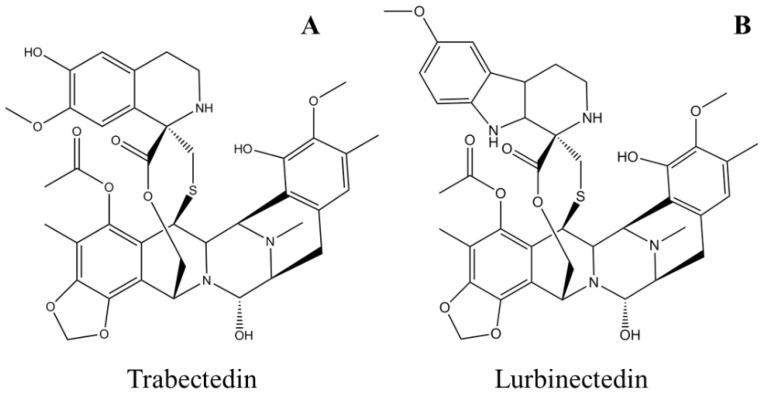
Schematic molecular representations of (**A**) trabectedin and (**B**) lurbinectedin.

Trabectedin, aka ET-743 and YONDELIS^®^ (PharmaMar SA, Madrid, Spain), (Table 1) is a synthetic antineoplastic alkylating agent originally extracted from the tunicate *Ecteinascidia turbinate* [119]. The molecule consists of three tetrahydroisoquinoline alkaloid moieties interconnected with each other in a nearly rigid and complex structure (Figure 5A) [120]. The natural abundance of the drug, which is as low as 1 g per 1000 kg of tunicate, does not meet the need for preclinical and clinical experiments, which forced chemists to find a route for its chemical synthesis. Total synthesis of trabectedin and lurbinectedin was accomplished in 26 individual steps with a yield of 1.6% [121,122]. The drug was designated as an orphan [123] and approved by the European Medicines Agency (EMA) in 2007 for the treatment of soft-tissue sarcoma [13,124] and in combination with pegylated liposomal doxorubicin (PLD) for the treatment of relapsed platinum-sensitive ovarian cancer [14,125]. Yondelis^®^ (PharmaMar SA, Madrid, Spain) was further approved by the US FDA in 2015 for the treatment of liposarcoma or leiomyosarcoma that is unresectable or has metastasized (Table 1) [126]. Several adverse events, which are classified as moderate to high but manageable, have been reported during clinical trials of trabectedin, such as neutropenic sepsis, rhabdomyolysis, hepatotoxicity, cardiomyopathy, capillary leak syndrome, and embryo-fetal toxicity, which should be monitored during treatment [126,127]. The drug gets metabolized to many different substituted compounds and is mostly excreted out in feces and urine; the half-life is reported to be about 26 h [128,129].

**Figure 6:**
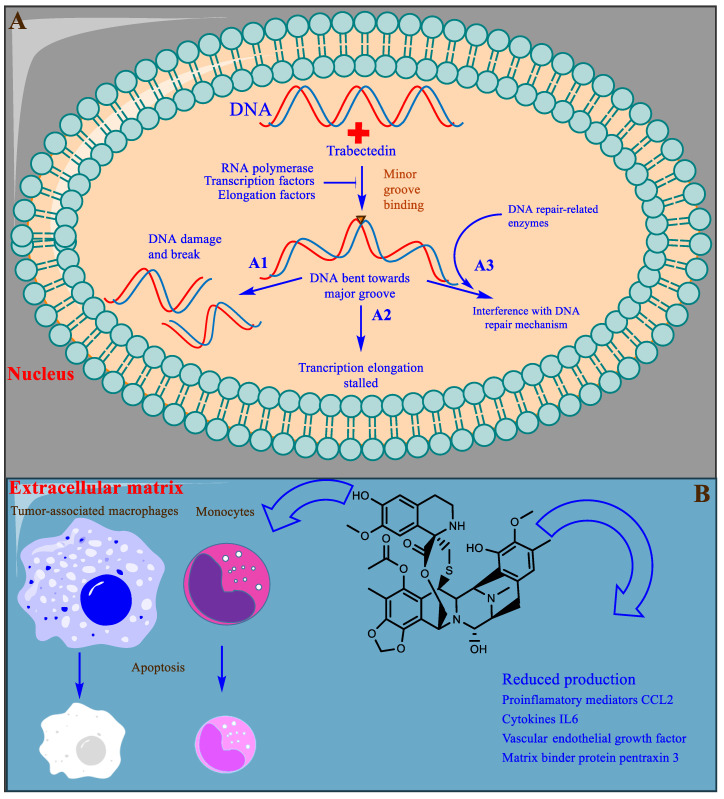
A representative illustration of the mechanism of trabectedin action. (**A**) The bilayer shows the nucleus, DNA strands are shown in red and blue stranded, and the inverted triangle in the minor groove represents trabectedin binding. (A1) DNA damage caused by trabectedin. (A2) The inhibition of transcription elongation by the inhibition of transcription factors and other related enzymes. (A3) The interference of trabectedin with the DNA repair machinery. (**B**) The extracellular matrix and the tumor microenvironment. TAMs and monocytes undergo cell death following inhibition by trabectedin, and trabectedin is shown in 2D in black stick representation.

Unlike most DNA alkylating agents, trabectedin binds to the minor groove guanine at the N2 position, initiating a cascade of events inducing cancer cell apoptosis. Trabectedin binding bends the DNA toward the major groove with drug protrusion on the convex side, and DNA subsequently loses affinity for its binding proteins, generating double-strand breaks [130,131,132] (Figure 6A). Specifically, trabectedin blocks cells in the G2–M phase of the cell cycle [133]. In addition to the general mechanism of cytotoxicity, trabectedin exerts atypical cell type-specific cytotoxicity. Cells deficient in nucleotide excision repair (NER) are 2–10 times less sensitive to trabectedin [134], whereas cells deficient in homologous repair (HR) are approximately 100 times more sensitive; however, in cells with nonhomologous end-joining (NHEJ) repair, no such differences were observed [135]. Trabectedin shows an atypical mechanism of the inhibition of tumor cell proliferation by interfering with the DNA repair machinery and inhibiting the active transcription process (Figure 6A).

Partial or indirect antitumor activity is also escalated by trabectedin following the disruption of the protumor functions of tumor-associated macrophages (TAMs) and monocytes present in the tumor microenvironment. TAMs produce many growth factors (GFs) (epidermal GF, fibroblast GF, and vascular endothelial GF), extracellular matrix-degrading enzymes, and immunosuppressive cytokines that are required for tumor proliferation and evasion of detection by the immune system [136]. Trabectedin shows selective cytotoxicity toward TAMs and monocytes, indirectly inhibiting tumor growth (Figure 6B) [137]. Trabectedin is unique among chemotherapeutic agents because it has multitargeted antineoplastic properties. It is also proposed to possess enormous therapeutic potential and is currently under experimental investigation [138].

#### 2.3.2. Lurbinectedin

Lurbinectedin, aka ZEPZELCA™ (PharmaMar SA, Madrid, Spain) is a synthetic analog of trabectedin in which the tetrahydroisoquinoline ring is replaced with tetrahydro β-carboline (Figure 5B) [139]. Lurbinectedin was granted orphan status and was approved for the treatment of adult metastatic small-cell lung cancer (SCLC) in 2020 by the US FDA (Table 1) [15,140]. Important adverse events observed in clinical trials were myelosuppression, hepatotoxicity, and embryo-fetal toxicity. Appropriate monitoring protocols should be implemented before and during drug administration, such as blood count and liver function tests [141,142]. The drug half-life (51 h) in the body increases when administered with CYP450 3A inhibitors, and the drug gets excreted from the body mostly in feces and urine [15,143].

The results of a clinical investigation of the effects of lurbinectedin on various tumors, such as SCLC, ovarian cancer, and breast cancer, indicated better pharmacokinetic profiles, which may allow the administration of higher therapeutic concentrations with less toxicity [144,145,146,147,148]. Lurbinectedin acts in a similar manner to trabectedin by inducing alkylation-dependent DNA damage, inhibition of active transcription, modulation of the DNA repair pathway, and modulation of the tumor microenvironment; however, monocytes are more sensitive to lurbinectedin [146,149]. The selective inhibition of RNA polymerase II transcription in SCLC has proven very effective [146], but another serious challenge is to counter the loss-of-function driver mutations in the tumor suppressor genes RB1 and TP53 and the amplification of the MYC gene. Targeted therapy and immunotherapy are anticipated to prolong the progression-free survival of patients with SCLC [150].

### 2.4. Antibody-Drug Conjugates

Antibody-based cancer therapy is one of the most advanced and successful techniques. The rationale for this approach is that the cell surface antigen phenotypes of tumor cells are different from those of their normal counterparts. Tumor cells selectively express, overexpress, or express mutated cell surface antigens that are selectively targeted by monoclonal antibodies (MABs). These MABs alone act as agonists or antagonists and neutralize enzymes after binding. MABs have also been conjugated with cytotoxins to selectively kill tumor cells, which are generally known as antibody-drug conjugates (ADCs) [151].

**Figure 7:**
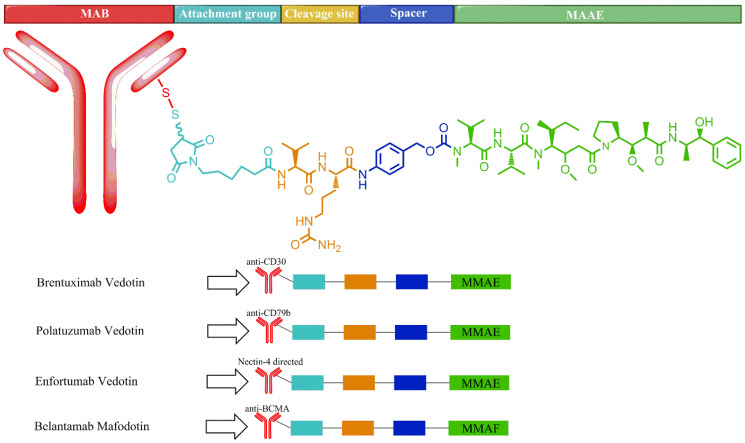
Sketch of an ADC. The antibody is connected to an attachment group that is further connected to an enzyme cleavage site. The drug is bonded to the cleavage site. Brentuximab vedotin differs from polatuzumab vedotin.

**Figure 8:**
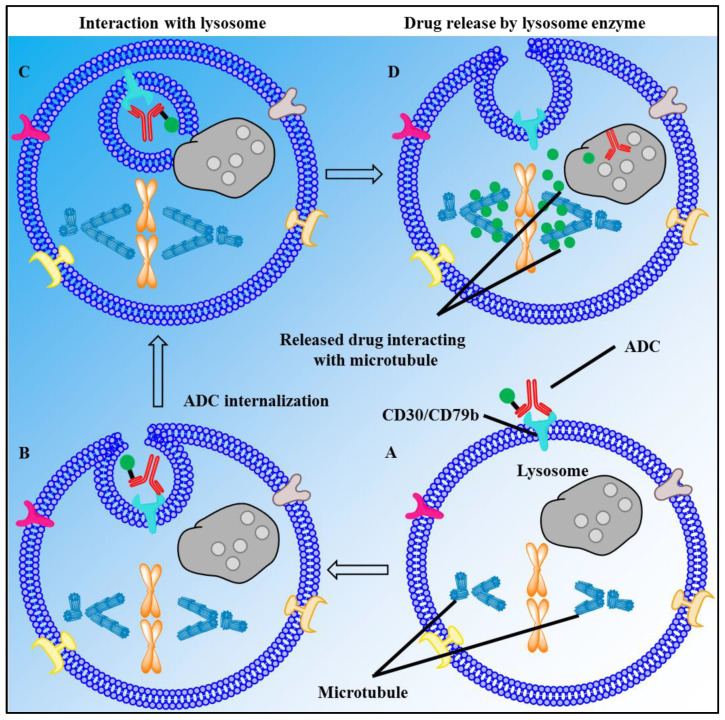
Schematic representation of the major mechanisms of action of brentuximab and polatuzumab: (**A**) binding of the ADC to the cell surface antigen (CD30/CD79b); (**B**) internalization of the ADC–cell surface antigen complex into the cell; (**C**) transportation of the ADC to the lysosome; (**D**) release of MMAE by lysosomal enzyme hydrolysis and the inhibition of tubule polymerization, resulting in apoptosis and cell death. The elimination of MMAE is mostly through CYP3A4/5 metabolic pathway and excretion via bile and feces. The half-life of MMAE is much shorter (~2.5 h) than that of the MMAE–antibody conjugate, which is 2.5–3 days [155].

ADC is a targeted cytotoxin delivery system that has been very successful, specifically in increasing drug concentrations in target cells, leading to apoptosis and cell death. Among many therapeutic agents, brentuximab vedotin (BV), polatuzumab vedotin (PV), and enfortumab vedotin (Padcev^®^, Astellas Pharma US Inc., Northbrook, USA) carry the marine-derived tubulin-binding drug monomethyl auristatin E (MMAE), and belantamab mafodotin (Blenrep^®^, GlaxoSmithKline, Brentford, UK) carries MMAF, which are linked to the specifically interacting MAB such as CD30, CD79b, nectin-4, and BCMA (B-cell maturation antigen), respectively (Figure 7) [152]. An average of 3.5 drug molecules bind on one antibody [153]. The drug is conjugated with the antibody through a hydrolyzable moiety along with other molecular moieties required for its stability (Figure 5). Following the binding of the MAB with the cell surface antigen, the ADC is internalized into the cell and transported to lysosomes for the release of the drug by hydrolysis. MMAE disrupts tubulin polymerization and stops cell division, resulting in apoptosis and cell death, while the MMAF payload binds to tubulin and stops the cell cycle at the DNA damage checkpoint between G2 and M phases, resulting in apoptosis (Figure 8) [151,154].

#### 2.4.1. Brentuximab Vedotin

Brentuximab Vedotin or BV, aka ADCERTIS^®^ (Seattle Genetics, Bossel, USA) is an anti-CD30 antibody-containing ADC approved by the US FDA in 2011 for the treatment of various T-cell lymphomas (Table 1). BV has been indicated for the treatment of adult patients with relapsed classic Hodgkin lymphoma (cHL), systemic anaplastic large-cell lymphoma (sALCL), primary cutaneous anaplastic large-cell lymphoma (pcALCL), or CD30 expressing mycosis fungoides (MF) [16].

CD30 is a cell surface glycoprotein that belongs to the tumor necrosis factor receptor superfamily (TNFRSF8, member 8). CD30 expression on normal cells is limited to a small population of activated B and T lymphocytes, natural killer cells, and some viruses (such as human immunodeficiency virus (HIV), T-lymphotropic virus-1 (HTLV-1), or Epstein-Barr virus (EBV))-infected cells. CD30 is also characteristically expressed in hematopoietic malignant cells in cancers such as cHL, sALCL, MF, and pcALCL [156,157]. The differential expression of CD30 on normal and hematopoietic malignant cells has directed researchers to explore the mechanism of action and its use in targeted cancer therapy. Chemotherapy, radiation therapy, and anti-CD30 antibody (alone/naked or conjugated with immunotoxin therapy) have a lower success rate in clinical trials than BV, which substantially prolongs progression-free survival [158].

Patients receiving BV require continuous observation, as various but mostly manageable toxicities have been observed, including peripheral neuropathy, anaphylaxis and infusion reactions, hematological toxicities, opportunistic infections, tumor lysis syndrome, hepatotoxicity, pulmonary toxicity, serious dermatological reactions, gastrointestinal complications, and embryo-fetal toxicity [16]. Freely circulating extracellular CD30-positive vesicles carrying ADCs may bind to CD30L-expressing cells, resulting in their death, which is probably the cause of off-target binding and toxicity (an in vitro study) [159]. BV has delivered better performance than the classical treatment regimen, but neuropathy and other adverse events may limit the long-term use of BV, which must be overcome by obtaining a better understanding of the role of CD30 in antiapoptotic mechanisms [157].

#### 2.4.2. Polatuzumab Vedotin

Polatuzumab Vedotin or PV, aka POLIVY™ (Genentech, San Francisco, CA, USA) is an anti-CD79b antibody-containing ADC approved by the US FDA and EMA in 2019 (Table 1) [17]. PV administered in combination with bendamustine and a rituximab product (BR) is used for the treatment of adult patients with relapsed or refractory diffuse large B-cell lymphoma (DLBCL). CD79b is expressed on most mature B cells; however, it is ubiquitously expressed on DLBCL cells, making it a logical target for disease treatment [160]. Other lymphomas, such as mantle cell lymphoma, Burkitt lymphoma, and follicular lymphoma (FL), have also been shown to express CD79b [161]. Among all non-Hodgkin lymphomas, 31% are DLBCL, and 50–70% are cured with BR treatment, but the disease relapses in a fraction of treated patients and has a dismal prognosis [162]. Similar to BV, PV also releases MMAE to cells expressing CD79b, a cell surface glycoprotein specific for PV (Figure 8) [160]. Compared to the classical treatment regimen with BR, PV in combination with BR produces a twofold higher rate of complete remission, which formed the basis of the approval of the latter treatment regimen [163]. Although treatment with PV promises better and longer, healthier lives, PV is associated with toxicity similar to that of BV and should be used with extreme care [160,164].

#### 2.4.3. Enfortumab Vedotin

Enfortumab vedotin or EV, aka Padcev^®^ (Astellas Pharma US, Inc., Northbrook, USA) is an anti-necrin-4 antibody-containing ADC approved by the FDA in 2019 for the treatment of advanced or metastatic urothelial cancer (Table 1) [18]. EV is attached to MMAE via a protease-cleavable linker [165]. It also contains a fully human monoclonal antibody directed against Nectin-4, an extracellular adhesion protein that is expressed at high levels in urothelial cancers [166]. Similar to PV, after the enfortumab–Nectin-4 complex is internalized into the cell, EV releases MMAE to cells expressing Nectin-4 through proteolytic cleavage [167]. Subsequently, the microtubule network within the cell is disrupted, arresting the cell cycle and inducing apoptosis [165]. EV is an anticancer agent that destroys tumor cells by inhibiting their ability to replicate but not in patients with a moderate to severe hepatic impairment [165]. In addition, EV may also cause significant hyperglycemia, leading, in some cases, to diabetic ketoacidosis, and it should not be administered to patients with a blood glucose level >250 mg/dL [165].

#### 2.4.4. Belantamab Mafodotin

Belantamab mafodotin or BM, aka Blenrep^®^ (GlaxoSmithKline, Brentford, UK) is an anti-B-cell maturation antigen (BCMA) antibody-containing ADC approved by the FDA in 2020 (Table 1) [19]. BM has been indicated for the treatment of adults with relapsed or refractory multiple myeloma who have received at least four prior therapies, including an anti-CD38 monoclonal antibody, a proteasome inhibitor, and an immunomodulatory agent [168]. It treats patients with the aforementioned characteristics through antibody-dependent cell-mediated cytotoxicity, G2/M cell-cycle arrest, and apoptosis [169]. BM is an afucosylated monoclonal antibody that targets BCMA, which is uniquely expressed on CD138-positive myeloma cells and conjugated to the microtubule disrupter monomethyl auristatin-F (MMAF) [170]. Its afucosylation of the Fc region of BM enhances antibody-dependent cell-mediated cytotoxicity [169,170]. Because of its risk of adverse effects and long duration of action, as it is administered every 3 weeks, BM shows a narrow therapeutic index [168]. Extreme care is essential due to the high risk of keratopathy that has been reported during clinical trials of BM, with a rate of approximately 71% [168].

### 2.5. Peptides or Proteins Used as Drugs or in Drug Preparations

Peptide or protein therapeutics from natural sources have increased in popularity in recent years because of their success in medicinal use. These molecules often have a high molecular mass, greater than 500 Da, and possess complex structures. These molecules usually selectively bind to other macromolecules or are efficient carriers for drugs or vaccines. To date, four marine-derived peptides, namely, plitidepsin, ziconotide, protamine, and keyhole limpet hemocyanin (KLH), have been identified. These molecules have been discovered from different sources and possess diverse therapeutic properties [152,171].

#### 2.5.1. Plitidepsin

**Figure 9:**
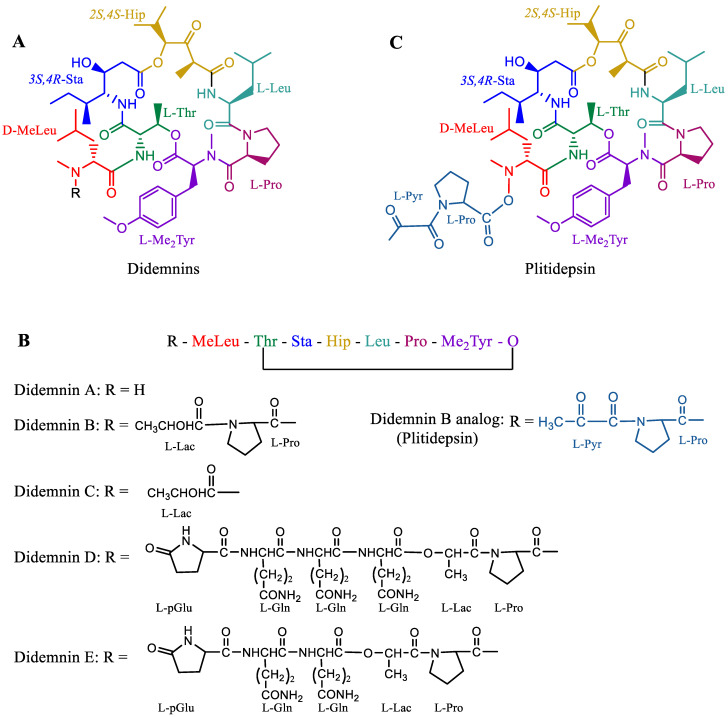
Molecules of the didemnin class. (**A**) General structure of didemnin, where “R” represents different substitutions in various didemnin members. (**B**) Simplified linear representation of a general didemnin. (**C**) Plitidepsin is similar to didemnin B, with a difference only in the terminal lactate that is oxidized to pyruvate. Short representations of residues: MeLeu—methyl leucine, Thr—threonine, Sta—statin, Hip—hydroxyisovalerylpropionic acid, Leu—leucine, Pro—proline, Me2Tyr—dimethyl tyrosine, Lac—lactate, Pyr—pyruvate, p-Glu—cyclic glutamate, and Gln—glutamine.

Plitidepsin, aka APLIDIN^®^ (PharmaMar SA, Madrid, Spain) is a synthetic cyclic peptide that was originally extracted from the ascidian *Aplidium albicans* (sea squirt). Plitidepsin belongs to a class of compounds known as didemnins (Figure 9A) [171]. Plitidepsin is an analog of didemnin B (Figure 9B,C). Other marine tunicates, such as *Trididemnum solidum*, are also abundant sources of similar depsipeptides, such as didemnins A–E (Figure 9B), which also possess antitumor and antiviral activities [172,173]. Plitidepsin was approved for the treatment of relapsed and refractory multiple myeloma in combination with dexamethasone by the Therapeutic Goods Administration (TGA), Australia, in 2018 [20]. The drug was granted orphan status by the EMA in 2003 as a treatment for leukemia, but the EMA did not approve the drug for relapsed and refractory multiple myeloma in combination with dexamethasone, citing that “the drug risk did not outweigh its benefits” [174]. Following the EMA refusal for marketing authorization of Aplidin, a lawsuit was filed by PharmaMar SA; the EU general court annulled the EMA decision, and the application was extended for review in October 2020 (Table 1) [174]. Recently, in preclinical studies, Aplidin was shown to be approximately 28 times more efficient than remdesivir against coronavirus disease 2019 (COVID-19) [175].

A phase III trial was conducted on the basis of the successful results from the phase II trial of Aplidin in patients with relapsed and refractory multiple myeloma. A significant improvement in the OS of the patients was reported for the drug in combination with dexamethasone (median OS of 11.6 months) compared to dexamethasone treatment alone (median OS 6.7 months) [176,177]. Most of the plitidepsin-related adverse events were manageable and well tolerated. Common adverse events reported were nausea, fatigue, and myalgia. Severe adverse events were related to hematological abnormalities such as anemia and lymphopenia that required continuous observation [178].

**Figure 10:**
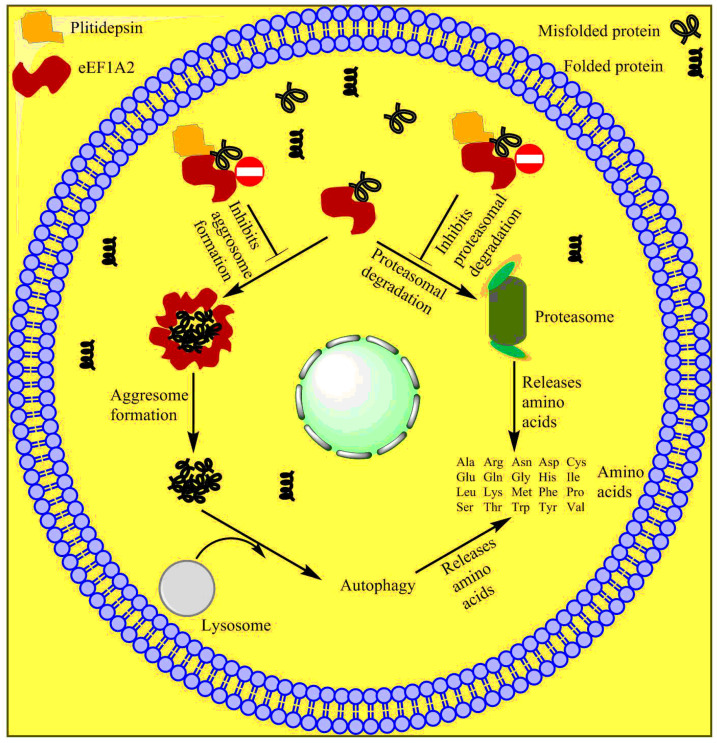
Mechanism of action of plitidepsin. Plitidepsin binds with eEF1A2 and blocks proteasomal aggresome degradation of misfolded proteins.

Multiple myeloma cells are often known to produce large amounts of misfolded proteins, which deplete free amino acids for new protein synthesis. The deposition of misfolded proteins is toxic to myeloma cells. These cancer cells devise mechanisms to destroy and recycle these misfolded proteins to obtain free amino acids and produce new proteins (Figure 10). Cereblon identifies misfolded proteins and redirects them for proteasomal degradation. The eEF1A2 protein, which is often overexpressed in myeloma cells, binds to misfolded proteins and redirects them for proteasomal degradation. When proteasomes do not function well or are inhibited, eEF1A2 forms a cluster of misfolded proteins after binding to them, which are subsequently destroyed by the aggresome during autophagy. Plitidepsin binds to eEF1A2 and renders it inefficient for binding with misfolded proteins and, hence, inhibits the destruction of misfolded proteins and cell apoptosis [179,180]. The tradeoff between the therapeutic efficiency and toxicity of the drug has remained controversial for a decade, which has kept researchers’ opinions split. However, the unique mechanism of the drug is promising for the treatment of multiple myeloma. Researchers have yet to discover appropriate combinations of drugs that may pave the way for the global clinical use of Aplidin.

#### 2.5.2. Ziconotide

**Figure 11:**
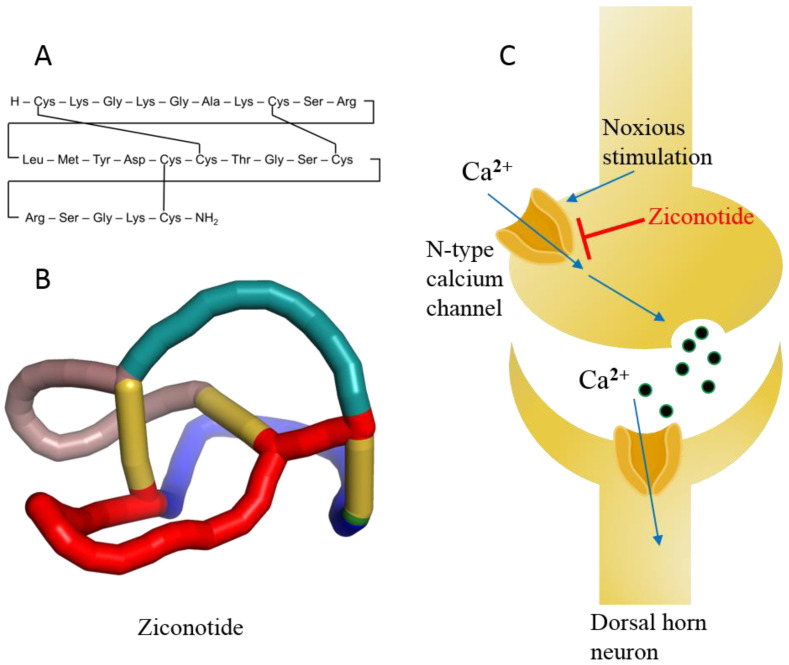
(**A**) Linear model of ziconotide with cross-linking of disulfide bonds. (**B**) A 3D knotted model of ziconotide. (**C**) Schematic diagram of ziconotide blocking the N-type calcium channel.

Ziconotide, aka PRIALT^®^, Azur Pharma International Limited, Dublin, Ireland) (Figure 11A,B) is a disulfide-rich and 25 amino-acid long conotoxin peptide (or conopeptide) that was originally extracted from the venom (i.e., a cocktail of toxic peptides) of the marine cone snail *Conus magus* and is used by the organism to hunt its prey and defend against predators [181]. More than 750 species of the genus *Conus* are known to produce 100s of toxic peptides in their venom, accounting for ~10,000 different conopeptides [182]. These toxins have attracted increasing interest among drug developers because of their magnificent potency and selectivity toward mammalian ion channels, G-protein-coupled receptors, transporters, and enzymes. Conotoxins are 10–40 amino acids long, mostly disulfide-rich (two to three disulfide bonds) and are classified on the basis of their sequence, disulfide framework or type of mammalian target they bind [183].

Ziconotide is the synthetic analog of ω-conotoxin and is highly soluble in water. In addition, ziconotide is the only nonopioid drug approved for the treatment of severe chronic neuropathic pain [184]. The drug is nonaddictive and is over 1000 times more effective than the classical opioid drug morphine, which is used for chronic pain. Ziconotide was approved in 2004 by the US FDA and in 2005 by the EMA (Table 1) [21]. Ziconotide is the only conotoxin approved for clinical use after three decades of research on the 1000s of conotoxins.

Ziconotide is an N-type voltage-gated ion channel blocker that inhibits the release of pro-nociceptive neurotransmitters (norepinephrine) and neuropeptides, relieving pain (Figure 11C) [21,185]. The median half-life of ziconotide in cerebrospinal fluid is approximately 4.5 h upon intrathecal injection, and it is completely cleared in 24 h. The metabolism of ziconotide is by endo- and exo-peptidases [186,187]. The most common adverse events reported during clinical trials are dizziness, nausea, nystagmus, confusion, gait abnormalities, memory impairment, blurred vision, headache, asthenia, vomiting, and somnolence [188]. Most of the adverse events associated with ziconotide monotherapy are mild to moderate and disappear over time [21,185,188]. The treatment of patients with chronic pain was also discontinued in some cases [189]. Therefore, experts believe that the psychological state of patients must be assessed a priori before treatment with ziconotide [190]. Ziconotide has been in clinical use for more than a decade and holds precedence for cancer-related and some noncancer-related pain management because of its high benefit-to-risk ratio compared to other opioid drugs, even today [191,192]. Experts also propose that this class of diverse peptide drugs may contribute to the list of therapeutic medicines for various diseases in the future because of its selectivity toward receptors, ion channels, and enzymes [184].

#### 2.5.3. Protamine Sulfate

Protamine sulfate is the oldest drug of marine origin approved by the US FDA and has been in clinical use since 1939 [22,23] for heparin overdose. Marketing authorization of protamine sulfate is held by many pharmaceutical companies under various trade names, such as Prosulf^®^ (CP Pharm and Wockhardt, Mumbai, India, Wales, UK) and Protam^®^ (Eipico, Ramadan City, Egypt) [193] (Table 1). Protamine sulfate is a strongly basic peptide containing more than two-thirds of the amino acid arginine. Protamine was originally obtained from the salmon sperm head, where it remained bound to negatively charged nucleic acids [194]. Protamine is 33 amino acids long and possesses the sequence MET PRO ARG ARG ARG ARG SER SER ARG PRO VAL ARG ARG ARG ARG ARG PRO ARG VAL SER ARG ARG ARG ARG ARG ARG GLY GLY ARG ARG ARG ARG (UniProt ID P69014). Because of its unique sequence, protamine may possess a random coil structure in solution or develop a secondary structure when bound to DNA. Positively charged arginine is present in four clusters, which prevents it from attaining a specific secondary structure because of strong charge repulsion [195]. Protamine is used as an antidote for heparin-induced anticoagulation in patients undergoing hemodialysis and heart surgery. Heparin neutralization usually occurs within 5 min of an intravenous protamine injection and is predominantly cleared from the body through urine [196].

**Figure 12:**
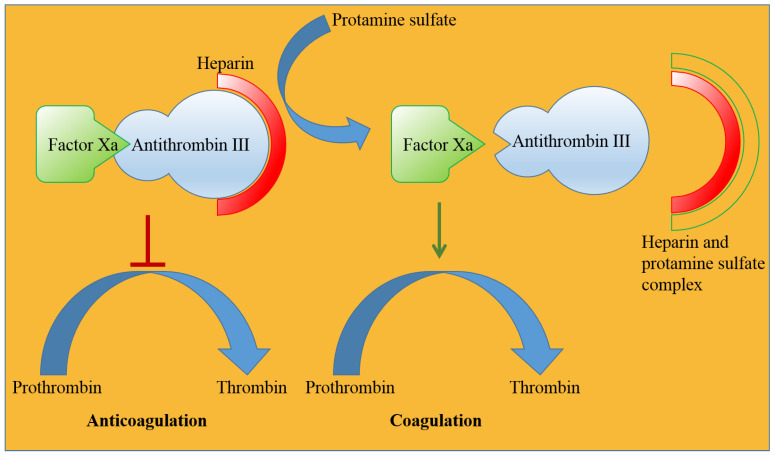
Cationic protamine sulfate reverses the anticoagulant effect of heparin by forming an ionic complex with heparin, which facilitates strong binding of factor Xa and weak binding of antithrombin and restores the natural coagulation process.

Blood coagulates at the site of injury through the activation of many coagulation factors and serine proteases, such as IXa, Xa, Xia, and XIIa, resulting in the conversion of prothrombin to thrombin that finally converts fibrinogen into fibrin, which subsequently forms a mesh-like structure (called a clot) [197]. Low-molecular-weight heparin (LMWH) binds antithrombin III (a serine protease inhibitor) and enhances its affinity for factor Xa, preventing the formation of thrombin, which ultimately inhibits fibrin formation at the site of injury [198]. Cationic protamine binds to anionic LMWH, rendering antithrombin III free and resulting in an intrinsic coagulation process (Figure 12) [199]. Protamine remains successful in removing heparin from plasma, but albumin-bound heparin is released over time and restores the anticoagulant effect. A high dose of protamine is often associated with adverse events in patients who have been administered heparin during heart surgery. Common adverse events associated with protamine use are allergic reactions, low blood pressure, dyspnea, nausea, vomiting, lassitude, and back pain, among others [193]. Although protamine has a satisfactory performance in many patients undergoing heart surgery, it is often associated with severe adverse events, which has reduced its market value. Active research is underway to identify less toxic and noninvasive alternative agents for anticoagulation reversal in addition to the approved drug Andexxa^®^ (Portola Pharmaceutical, South San Francisco, USA in 2018) [200]. Many more protamine alternatives are expected to be developed in the near future.

#### 2.5.4. Keyhole Limpet Hemocyanin (KLH)

**Figure 13:**
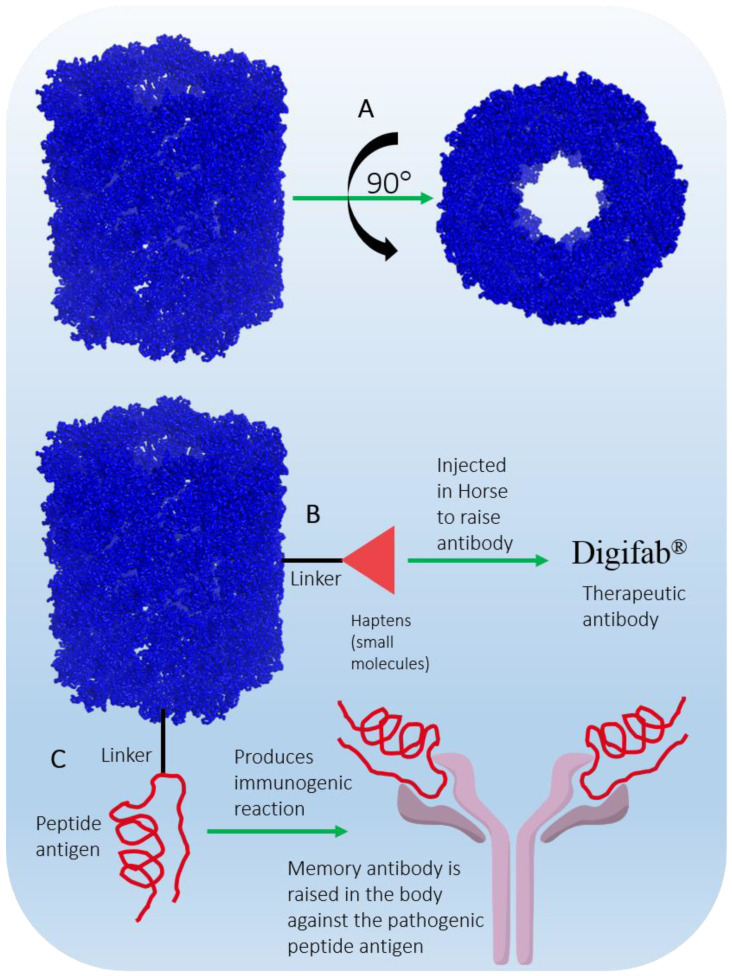
(**A**) KLH barrel-shaped representation drawn using cryo-electron microscopy 3D coordinates (PDB ID 4BED). (**B**) KLH as a hapten conjugate. (**C**) KLH as a vaccine carrier.

KLH is a large barrel-shaped, multi-subunit, oxygen-carrying metalloprotein present in the hemolymph of the marine mollusk *Megathura crenulata* (Figure 13A and Table 1) [201]. KLH in itself is not a drug, but it is approved (as mentioned in DrugBank) for the preparation of therapeutic agents for the treatment of cancer and other immunological diseases by eliciting an appropriate immune response in human patients [202]. KLH is an immunomodulator and has made a valuable contribution to immunological research and the global immunopharmaceutical market for more than 50 years since its astounding immunostimulatory properties were documented in experimental animals and even in humans [203,204,205,206].

KLH is present in two isoforms, KLH1 and KLH2, with monomeric molecular masses of 390 kDa and 360 kDa, respectively [207]. KLH is composed of 20 monomers, where each monomer contains 7–8 functional domains. Each domain contains two copper ions (Cu^2+^) and binds one O_2_ molecule. The gigantic didecameric structure is heavily glycosylated, which makes it suitable for desirable hapten conjugation (Figure 13B), and it is used as a vaccine carrier molecule (Figure 13C). Due to its complex glycosylation and large size, laboratory synthesis is nearly impossible; therefore, commercial production is achieved by purification from the hemolymph of the animal [208]. KLH is immunogenic but does not elicit adverse immune responses in humans; hence, it is safe to use as an immunomodulator [209]. Immucothel^®^ from Biosyn Corporation is a commercial subunit product that has been used as a building block for the reassociation of KLH in the presence of appropriate ions. Immucothel^®^ was approved in 1997 for the treatment of bladder cancer and is marketed in the Netherlands, Austria, Argentina, and South Korea [24,209]. Vacmune^®^ is another immunocyanin commercial product from Biosyn Corporation used as a protein carrier for vaccine development (Figure 13B and Table 1) [25]. Commercially available KLH is often used to generate antibodies against small molecular moieties present in large complexes. The antibodies generated have been used as therapeutic agents in the production of the approved and marketed antibody drug Digifab^®^ (ovine Digoxin Immune Fab), BTG International Inc., (London, UK) 2017 (Figure 13B) [210]. Other commercial products generated from KLH that have been used to analyze antibody titers in various immunological assays are ImmuneActivator™ (PerImmune, Rockville, MD, USA), KLH-ImmuneActivator^®^ (Organon Technica, Durham, USA), and ImmuneActivator™ (Intracel, Rockville, MD, USA). A more comprehensive list of commercial KHL products used for immunotoxicological studies has been discussed in detail by Swaminathan et al. [211]. Active research to explore and optimize the immunostimulatory and immunomodulatory properties of KLH, which are of a very complex nature, may achieve therapy for diseases such as cancer in the future.

### 2.6. Fish Oil and Its Components

**Figure 14:**
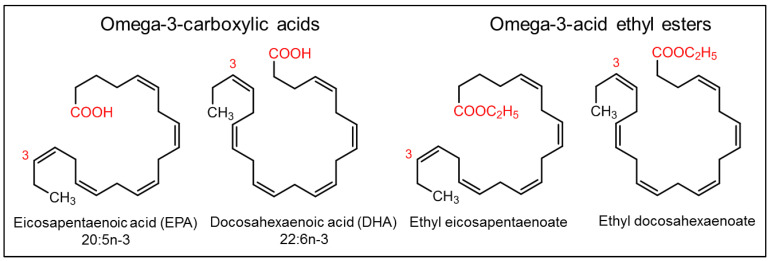
Omega-3 fatty acids and their derivatives.

Fishes are well known for their health benefits worldwide. Fish accumulate essential fatty acids such as omega-3 fatty acids (primarily synthesized in marine algae) in their body through the marine food chain. Epidemiological studies have shown that people who consume fish regularly are at lower risk of coronary heart disease mortality than those who consume fish less often or consume none [212,213,214,215,216]. The health benefits associated with omega-3 fatty acids are mainly attributed to (a) alpha linolenic acid (ALA, C18:3, *n*-3), mostly of plant origin, (b) eicosapentaenoic acid (EPA, C20:5, *n*-3), which are mostly of marine origin, and (c) docosahexaenoic acid (DHA, C22:6, *n*-3), which are mostly of marine origin (Figure 14).

Many of the fish oil components and their derivatives are approved by the US FDA as nutraceuticals and are commercially available on the market today (Table 1, Table 2 and Figure 14) [217], including (a) omega-3-acid ethyl esters, marketed as Lovaza^®^ (2004, GlaxoSmithKline, Brentford, UK) and in European countries as Omacor^®^ (2004, GlaxoSmithKline, Brentford, UK) and Omtryg^®^ (2004, Trygg Pharma, Oslo, Norway), (b) icosapent ethyl, marketed as Vascepa^®^ (2012, Amarin Pharma, Inc., Dublin, Ireland), (c) omega-3-carboxylic acids, marketed as Epanova^®^ (2014, AstraZeneca Pharmaceuticals LP, London, UK), and (d) fish oil triglycerides, marketed as Omegaven^®^ (2018, Fresenius Kabi, Fresenius Kabi, Bad Homburg, Germany) [218,219].

**Figure 15:**
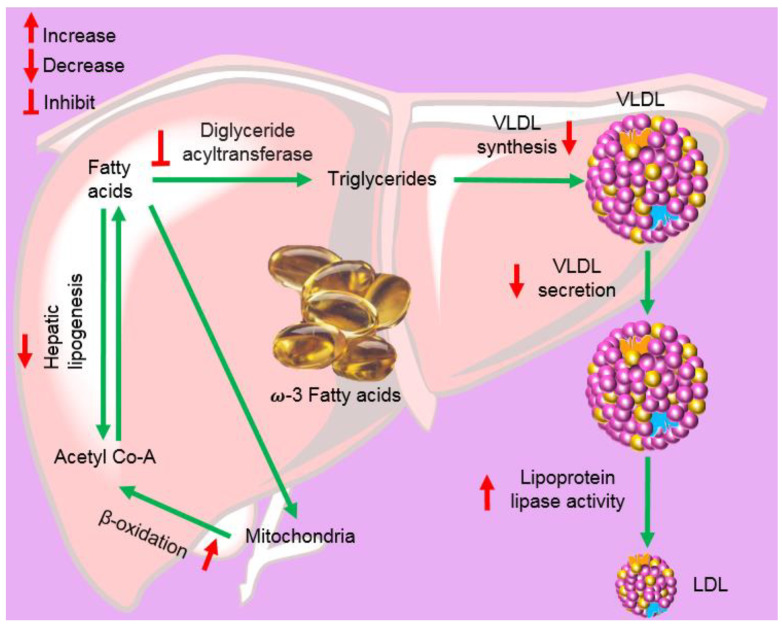
Mechanism via which omega-3 fatty acids lower triglycerides and the conversion of very-low-density lipoprotein (VLDL) into low-density lipoprotein (LDL).

Omega-3-acid ethyl esters, icosapent ethyl, and omega-3-carboxylic acids are all approved for the treatment of hypertriglyceridemia (triglycerides > 150 mg/dL) and severe hypertriglyceridemia (triglycerides > 500 mg/dL) [220,221,222]. These substances differ in the form of fatty acids and their amount in each 1 g capsule. Lovaza^®^, Omacor^®^, and Omtryg^®^ contain a mixture of ethyl esters of EPA and DHA. Vascepa^®^ contains the ethyl ester of EPA as a major ingredient [222]. Epanova^®^ contains a mixture of carboxylic acids of EPA and DHA. The bioavailability of forms of EPA and DHA was highest in Vacepa^®^ (Table 2). Other inactive ingredients are also present in these formulations, such as tocopherol, gelatin, glycerin, maltitol, sorbitol, and purified water. These formulations help to reduce triglyceride and very-low-density lipoprotein levels (Figure 15). The mechanism underlying these effects is not very well understood, but the potential mechanisms proposed to date include the inhibition of diacylglycerol acyltransferase, increased activity of plasma lipoprotein lipase, a decrease in lipogenesis, and an increase in β-oxidation in the liver (Figure 15) [218,223].

Omegaven^®^ is a fish oil-based emulsion approved for the treatment of parenteral nutrition-associated cholestasis (PNAC) in pediatric patients as a source of calories and fatty acids. Major risk factors for the development of PNAC are premature birth and low birth weight [224]. The drug is administered intravenously as an emulsion injection. The drug is not known to prevent PNAC. The mechanism underlying the success of the treatment is unknown, as researchers have not determined whether the anti-inflammatory effect or the effect of the ratio of omega-3/omega-6 fatty acid components in the drug is responsible. Experts recommend routine laboratory tests to monitor the levels of essential fatty acids when treating patients, as they often drop below normal levels [225].

## 3. Discussion

We can learn great scientific lessons from nature’s design of diverse molecules, through which they produce specific functional, mechanical, and enzyme-inhibitory effects, with biocompatibility and immunological properties in human systems. These successful molecular designs attain molecular complementarity for the site of action on proteins and nucleic acids without compromising the thermodynamic affinity. Small marine compounds such as spongonucleotides and long-chain omega-3 fatty acids may have achieved success because of their ability to mimic their natural counterparts. The selectivity of large marine compounds such as trabectedin, ziconotide, and plitidepsin correlates well with their large size, nearly rigid conformation, and specific topology. BV and PV are excellent examples of the targeted delivery of cytotoxic agents. Positively charged protamine sulfate efficiently sequesters negatively charged heparin through strong ionic interactions. KLH elicits an immunological response because of its large size, complex posttranslational modifications, and immunocompatibility. All these diverse factors that led to the development of successful drugs must be emphasized in future drug design. Inspired by the discovery of these successfully marketed drugs and their diversity in structure and function, we could also use their valuable and specific structural backbones to further optimize and design a series of better marine drugs. By maintaining the skeletal structure of trabectedin and replacing the tetrahydroisoquinoline ring with tetrahydro β-carboline, the derivative lurbinectedin shares a similar mechanism of action with trabectedin and selectively inhibits RNA polymerase II transcription in SCLC [226]. In conclusion, these factors will help inspire researchers to explore all possible combinations of factors for novel drug design rather than to limit themselves to traditional drug characteristics.

Most of the approved marine drugs discussed above are reserved for the cure of various cancers [227,228,229,230,231], but the mechanism of action is quite diverse. For example, spongonucleosides (CYTOSAR-U^®^, DEPOCYT^®^, VIRA-A^®^, FLUDARA^®^, ARRANON^®^, and ATRIANCE^®^) act as antimetabolites, EM (HALAVEN^®^) is a microtubule inhibitor, trabectedin (YONDELIS^®^) and lurbinectedin (ZEPZELCA™) are DNA alkylating agents, BV (ADCERTIS^®^) and PV (POLIVY™) deliver MMAE to specific cell types, and plitidepsin (APLIDIN^®^) prevents protein recycling by binding to eEF1A2. Other approved marine drugs are clinically used to treat chronic neuropathic pain (ziconotide, i.e., PRIALT^®^) and heparin overdose during surgery (protamine sulfate) or as an immunomodulatory agent or vaccine carrier (KLH). Nutraceuticals that contain omega-3 fatty acids and their derivatives (LOVAZA^®^, EPANOVA^®^, VASCEPA^®^, and OMEGAVEN^®^) as major components from fish help to reduce circulating triglyceride contents in blood and prevent coronary heart disease-related mortality.

**Figure 16:**
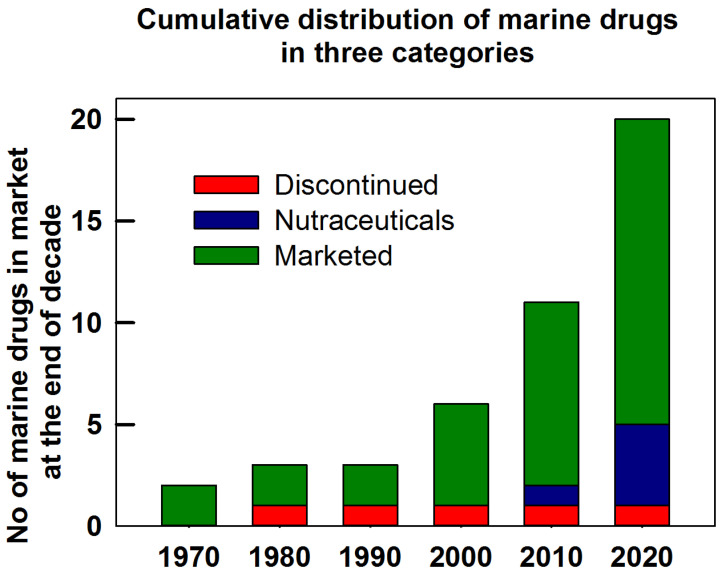
Cumulative number of marine drugs available on the market at the end of consecutive decades from 1970 to 2020.

As shown in Figure 16, the considerable increase in the availability of marine drugs for clinical use over the past six decades is an indication not only of the popularity of marine compounds for the treatment of existing and neglected diseases among researchers and experts but also of people’s faith in the clinical use of marine drugs, suggesting a very high potential for marine compounds to be developed into clinical drugs [232]. Jimenez et al. noted that various marine organisms or microorganisms, such as sponges, mollusks, fungi, bacteria, sea urchins, and microalgae, produce different types of therapeutic marine-derived compounds, showing promising prospects for marketed drugs [233]. Galasso et al. explored the current roles of marine microalgae as proposed ingredients for functional foods that possess an advantage in their potential chemopreventive properties [234], while the review by Shikov indicated that the unique naphthoquinone pigment from sea urchins possesses diverse chemical and pharmacological properties [235]. In 2020, Wei et al. reported that certain marine compounds exert antineoplastic effects on various cancers, including lung cancer, breast cancer, and prostate cancer, through important signaling pathways, such as the PI3K/Akt and MAPK signaling pathways [236,237]. Due to their unique skeletal structures, these marine compounds are very promising for cancer research after being optimized by certain computer-aided drug design pipelines. Scientists and researchers are optimistic about the availability of unique scaffolds in natural products that could treat many more diseases. However, this task will be difficult without an efficient method for determining the corresponding targets of each marine product, which is currently a major bottleneck for marine compound development into drugs and one of the main reasons for the side-effects and adverse reactions of marine compounds.

Nevertheless, scientists worldwide are constantly working hard to explore the tremendous amount of potential of marine natural products to become therapeutic clinical candidates [238,239,240]. Some drugs have achieved rapid progress and have been approved for marketing in recent years or entered clinical trials for other indications [241,242]. Currently, many marine natural products are in various stages of clinical investigation, as shown in Table 3. Because of the success of the ADC strategy to neutralize specific cancer cells, many other cell surface receptors are being studied for targeting and killing cancer cells with greater specificity. In addition, most of the marine drugs being investigated in clinical trials exert therapeutic effects on cancer, indicating the huge potential for the development of numerous cancer drug candidates.

Readers can refer to references [234,235] for a more comprehensive list of potential marine-derived compounds that may become future drugs. Other important reviews published can also be considered to obtain better knowledge of drugs used in the clinic, as well as those being investigated in clinical trials [238,241]. Here, in the present review, we compiled the most comprehensive list of marine-derived drugs approved for clinical use in at least one country and illustrated the mechanisms of action of these drugs.

However, many factors restrict the clinical promotion and application of drugs, one of which is serious adverse events. The side-effects of some drugs in clinical trials have hindered their further development; however, in some cases, they have promoted research on synthetic marine derivatives. For example, as listed in Table 3, soblidotin and synthadotin used in phase II clinical trials as treatments for various cancers are derivatives of marine dolastatin 10 and dolastatin 15, respectively [243]. In addition, the adverse reactions associated with some marketed marine drugs are mild and moderate but manageable, while those of others are severe and fatal, requiring real-time monitoring. In particular, neurological adverse events and other serious adverse reactions observed during nelarabine, EM, BV, and PV treatment discussed above require patients to be continuously observed. In addition to the specific target, a drug may interact with many other proteins that are considered off targets [244]. In most cases, off-target adverse drug reactions (ADRs) are usually different from common adverse reactions and cannot be predicted according to their discovered pharmacological action [245,246]. For example, an in vitro study indicated that BV may cause the death of cells that express the CD30L molecule because of off-target binding and toxicity. Therefore, the potential targets of action, off-target activities, and off-target ADRs must be identified for careful evaluations of these “off-target” effects, widening the therapeutic applications of drugs [246].

Traditional approaches to identifying targets include biochip [247,248], gene silencing [249], and gene knockouts [250,251], with relative disadvantages such as a high cost and low efficiency. Recently, the vigorous development of computational approaches has facilitated the faster discovery of drug-related targets, including potential therapeutic targets and off-targets, accelerating research progress in possible mechanisms of action and adverse reactions. Reverse screening is an effective method that identifies potential or unexpected targets for a particular molecule from among many receptors by examining the structures of known ligands or crystal structures to identify drug targets more efficiently [252]. This approach consists of three steps, namely, shape screening, pharmacophore screening, and reverse docking, and plays a crucial role in the discovery of targets and the study of molecular mechanisms [252]. After identifying targets, we can simulate the interaction of compounds and targets via computer-mediated molecular docking to acquire docking position models, providing a basis to improve the modification and optimization of marine drugs [253]. The use of computer-aided virtual screening [254], reverse target fishing [252,253], core hopping, and full chemical synthesis [255,256] can accelerate the pace of the identification of targets and the optimization of the skeleton to reduce adverse reactions. Combining drugs is also a major strategy for improving therapeutic efficiency and decreasing adverse effects. As shown in Figure 16, among the marketed marine drugs, an increasing number of nutraceuticals have been identified, which may accelerate the treatment processes of some diseases. For example, some fish oil components have the potential to treat patients with hypertriglyceridemia by inhibiting diacylglycerol acyltransferase, increasing the activity of plasma lipoprotein lipase, decreasing lipogenesis, and inducing β-oxidation processes in the liver. Marine drugs and their synthetic derivatives, whether used alone or in combination with other drugs, might provide new insights into clinical treatments in the future.

Among many experimental and investigational drugs of marine origin, very few have been approved for clinical use, as discussed in this review. Although scientists have achieved progress in technology and obtained a better understanding of human diseases, the development of novel drugs is still difficult because of the high cost and failure rate [257]. Combined with the target identification and structural modification strategy discussed above, drug repurposing is now a universal strategy for clinical drugs, with the advantage of saving time and cost compared to traditional de novo drug development approaches [258], especially during sudden epidemics such as COVID-19. As shown in Table 1, most of the marketed marine drugs were approved to treat more than one disease, most of which were discovered after years of research. For instance, the first approved marine drug, cytarabine, was authorized by the FDA to induce remission of acute nonlymphocytic leukemia in 1969 and for the treatment of lymphomatous meningitis in 1999.

At present, most marketed drugs of marine origin are still undergoing biomedical experimental investigations to find prospective new therapeutic targets, indications, and adverse events. One of the marketed drugs, APLIDIN^®^, has previously been approved for clinical use in cancer treatment, including pancreatic, stomach, bladder, and prostate cancers [20], while most recent preclinical studies have shown promising effects of APLIDIN^®^ in treating COVID-19 [175]. Additionally, as shown in Table 3, approved drugs such as lurbinectedin and plitidepsin are being further investigated for other indications because of their success in the cases examined and their potential success in new indications of diseases [140,238], suggesting that the drug repurposing strategy holds great mining prospects. Despite the large number of diverse studies on marine natural compounds [239], we focused our discussion in the respective sections of this review on the mechanisms of action and the indications for which the drug is approved. On the basis of in-depth scientific research and the improvement of science and technology, we may now discover new indications of approved drugs not only using experimental approaches, including binding assays to identify target interactions and phenotypic screening, but also through computational approaches, including signature matching, computational molecular docking, genome-wide association studies, pathway or network mapping, and retrospective clinical analysis [257]. We hope that the discussion presented within this review will drive the development of new marine drugs from marine compounds and the repositioning of existing marine drugs.

## 4. Materials and Methods

A literature search of the PubMed database was conducted before 30 June 2022. The main literature reviewed in this article was first retrieved with the following keywords: #1, ((marine*) OR (sea)) OR (ocean), #2, (((drug*) OR (product*)) OR (compound*)) OR (derivative*)) OR (agent*), #3, (clinical*) OR (clinic*), #4, FDA, #5, “1970/01/01” [Date–Publication]: “2022/06/30” [Date–Publication]. As shown in Table S1, the search results were integrated by the “AND” symbol of Boolean Calculation Search, with a retrieval statement of #1 AND #2 AND #3 AND #4 AND #5. The detailed literature search strategy and a flow diagram of the process used to select eligible studies are shown in Table S1 and Figure S1, respectively. In addition, we conducted a manual search of references included in articles on this topic to avoid missing related publications. The retrieved results were screened to eliminate articles with little relevance to marine products based on the title, keywords, and abstract. After further reading the full-text articles, the other studies not included in the total number of references mainly described the mining, extraction, synthesis, and analysis technology in the marine environment but did not describe the marketed marine drugs.

Studies were included in our review if they (1) stated marine products and (2) discussed marketed drugs used in the clinic. The exclusion criteria were (1) abstracts, editorials, communication, and unrelated reviews, (2) studies unrelated to marine products, (3) reports unassociated with marketed drugs, and (4) studies with few pharmacological data or mechanisms of action. We extracted the following information from each included study: the name of the marketed drug, brand name, marine source organism, structure type, mechanism of action, treatment indications, approving agency, and approval year.

## 5. Conclusions

Marine natural products are potent and promising sources of drugs among other natural products of plant, animal, and microbial origin. In this article, we reviewed six types of marine drugs, including 20 in clinical use, and discussed their diversity in terms of structures, mechanisms of action, and clinical indications. Most approved marine compounds are antineoplastic, but some are also used for chronic neuropathic pain, for heparin overdosage, as haptens and vaccine carriers, and for omega-3 fatty acid supplementation in the diet. At the same time, clinical trials of marine compounds with diverse skeletons and mechanisms have achieved great progress, inspiring new indications or derivative drug searches. In addition, we discussed these marine compounds in terms of the search for their targets and indications and the reduction in adverse events after elucidating their detailed mechanisms. Our article provides basic information to readers who are interested or majoring in the drug design field with hopes of designing more effective marine drugs for use worldwide or promoting the new usage of marketed marine drugs for human health to solve broader areas of disease-related complications.

Major success has been achieved in the development of cancer- and ADC-related drugs. However, other classes of compounds have also shown their potential in the respective fields, such as severe chronic pain, eye-related cellular degeneration, myocardial ischemia/reperfusion injury, heparin overdose, PNAC, and complications related to reduced triglyceride levels. The knowledge obtained from a large amount of literature was compiled here to provide readers up-to-date knowledge of marine drugs in clinical use. However, the marine drugs undergoing clinical investigation discussed in Table 3 provide readers with a comprehensive resource of valuable information on marine drug candidates.

## Figures and Tables

**Table 1:** Marketed drugs of marine origin along with their trade name, source of origin, mechanism of action, and disease treatment indications.

Name (DrugBank ID)	Brand Name (Company)	Source Organism	Type (MW)	Mechanism of Action	Treatment Indications, Approving Agency (Year)
**Spongonucleosides**					
**Cytarabine, ara-C** **(DB00987)**	CYTOSAR-U^®^ (Pfizer, New York City, NY, USA) and DEPOCYT^®^ (Pacira Pharma, San Diego, CA, USA; Bedford Lab, Seattle, WA, USA, Enzon Pharmaceuticals, Piscataway, NJ, USA)	*Cryptotethia crypta* (sponge)	Small molecule (243.22 Da)	Synthetic spongonucleoside analog, arrests cells in S phase by inhibiting DNA synthesis	Remission induction in acute nonlymphocytic leukemia, FDA (1969) [5] Lymphomatous meningitis, FDA (1999) [6]
**Vidarabine, ara-A** **(DB00194)**	VIRA-A^®^ (King Pharmaceuticals, Bristol, FL, USA)	*Cryptotethia crypta* (sponge)	Small molecule (267.24 Da)	Synthetic spongonucleoside analog, stops the replication of herpes viral DNA	Acute keratoconjunctivitis and recurrent superficial keratitis caused by HSV-1 and HSV-2, FDA (1976) [7]
**Fludarabine, F-ara-A** **(DB01073)**	FLUDARA^®^ (Sandoz, Basel, Switzerland) and OFORTA^®^ (Sanofi-Aventis, Paris, France)	*Cryptotethia crypta* (sponge)	Small molecule (285.23 Da)	Synthetic spongonucleoside analog, inhibits DNA synthesis by inhibiting DNA polymerase alpha, ribonucleotide reductase, and DNA primase	B-cell CLL, FDA (1991) [8]
**Nelarabine** **(DB01280)**	ARRANON^®^ (GSK, Brentford, UK) ATRIANCE^®^ (Novartis, Basel, Switzerland)	*Cryptotethia crypta* (sponge)	Small molecule (297.27 Da)	Synthetic spongonucleoside analog, is metabolized into ara-GTP, competes with dGTP, and is incorporated into the DNA, inhibiting DNA elongation	T-cell acute lymphoblastic leukemia and T cell lymphoblastic lymphoma, FDA (2005) [9]
**Histochrome**	Pacific-Ocean Institute of Bioorganic Chemistry, Vladivostok, Russia	*Scaphechinus mirabilis* (sea urchin)	Small molecule (220 Da)	The drug prevents DNA damage and regulates apoptosis under oxidative stress condition	Used to treat degenerative diseases of the retina and cornea, macular degeneration, etc., myocardial ischemia/reperfusion injury etc., Russia (1999) [10]
**Microtubule Inhibitors**					
**Eribulin mesylate (DB08871)**	HALAVEN^®^ (Eisai, Bunkyo, Japan)	*Halichondria okadai* (sponge)	Small molecule, orphan ^Ϯ^ (826.00 Da)	Polyether macrolide, arrests cells in G2/M phase by inhibiting microtubule growth after direct interaction	Metastatic breast cancer, FDA (2010) [11], unresectable or metastatic liposarcoma, FDA (2016) [12]
**DNA alkylating agent**					
**Trabectedin, ET-743 (DB05109)**	YONDELIS^®^ (PharmaMar SA, Madrid, Spain)	*Ecteinascidia turbinata* (tunicate)	Small molecule, orphan ^Ϯ^ (761.80 Da)	DNA alkylating agent, forms adducts with DNA guanine residue in the minor groove, bends the DNA helix toward the major groove, and disrupts the association of DNA binding proteins	Soft-tissue sarcoma and relapsed platinum-sensitive ovarian cancer, EMA (2007), [13] unresectable or metastatic liposarcoma or leiomyosarcoma, FDA (2015) [14]
**Lurbinectedin (DB12674)**	ZEPZELCA™ (PharmaMar SA, Madrid, Spain)	*Ecteinascidia turbinata* (tunicate)	Small molecule, orphan ^Ϯ^ (784.90 Da)	DNA alkylating agent, forms adducts with DNA guanine residue in the minor groove, bends the DNA helix toward the major groove, and disrupts the association of DNA binding proteins	Metastatic SCLC, FDA (2020) [15]
**Antibody-Drug Conjugates**					
**Brentuximab vedotin (DB08870)**	ADCERTIS^®^ (Seattle Genetics, Bothell, Wahington, WA, USA)	*Dolabella auricularia* (mollusk)	Biotech (~153 kDa)	The antibody component (IgG1) targets CD30 and MMAE disrupts the microtubules after internalization	Hodgkin lymphoma and systemic anaplastic large-cell lymphoma, FDA (2011) [16]
**Polatuzumab vedotin (DB12240)**	POLIVY™ (Genentech, San Francisco, CA, USA)	*Dolabella auricularia* (mollusk)	Biotech (~150 kDa)	The antibody component targets CD79b and MMAE disrupts the microtubules after internalization	Relapsed or refractory diffuse large B-cell lymphoma, FDA (2019) [17]
**Enfortumab vedotin (DB13007)**	Padcev^®^ (Astellas Pharma US Inc., Northbrook, IL, USA)	*Dolabella auricularia* (mollusk)	Biotech (~153 kDa)	The antibody component (IgG1) targets nectin-4 and MMAE disrupts the microtubules after internalization	Treatment of patients with advanced, treatment-resistant urothelial cancer, FDA (2019) [18]
**Belantamab mafodotin (DB15719)**	Blenrep^®^ (GlaxoSmithKline, Brentford, UK)	*Dolabella auricularia* (mollusk)	Biotech (~153 kDa)	The antibody component (IgG1) targets BCMA (B-cell maturation antigen) and MMAF disrupts the microtubules after internalization	Treatment of adult patients with relapsed or refractory multiple myeloma who have received at least 4 prior therapies, including and anti-CD38 monoclonal antibody, a proteasome inhibitor, and an immunomodulatory agent, FDA (2020) [19]
**Peptides or Proteins Used as Drugs or in Drug Preparations**					
**Plitidepsin (DB04977)**	APLIDIN^®^ (PharmaMar SA, Madrid, Spain)	*Aplidium albicans* (sea squirt)	Small molecule, orphan ^Ϯ^ (1110.30 Da)	Binds to the gene product of eEF1A2, thus inhibiting cancer cell viability	Tumors in pancreatic, stomach, bladder, and prostate cancers, TGA (2018) [20]
**Ziconotide (DB06283)**	PRIALT^®^ (Azur Pharma, Dublin, Ireland)	*Conus magus* (marine snail)	Small molecule (2639.20 Da)	Blocks excitatory neurotransmitter release by inhibiting the N-type calcium channels of primary nociceptive afferent nerves and relieves pain	Severe chronic pain, FDA (2004) and EMA (2005) [21]
**Protamine sulfate (DB09141)**	PROSULF^®^ (CP Pharm, Hong Kong, China; Wockhardt Mumbai, India; etc.) PROTAM^®^ (Eipico, Ramadan City, Egypt)	Salmon sperm heads	Biotech (~4.3 kDa)	Reversal of the anticoagulant effect of heparin by forming an inactive complex with heparin	Heparin overdose, FDA (1939) [22,23]
**Keyhole limpet hemocyanin (DB05299)**	IMMUCOTHEL^®^ VACMUNE^®^ (Biosyn Corporation, Carlsbad, CA, USA)	Keyhole limpet (marine mollusk)	Biotech (350 to 390 kDa)	An immunomodulator	IMMUCOTHEL^®^ for bladder cancer, [24], VACMUNE^®^ as protein carrier for vaccine development [25], the Netherlands (1997), Austria and South Korea (2001), Argentina (2004/2005) [26]
**Fish Oil and its Components Used as Drugs**					
**Omega-3-acid ethyl esters (DB09539)**	LOVAZA^®^ (GSK, Brentford, UK), OMACOR^®^ (GSK, Brentford, UK), and OMTRYG^®^ (Trygg Pharma, Oslo, Norway)	Fish	Small molecule (330.51 to 356.55 Da)	Reduces triglyceride synthesis by inhibiting 1,2-diacylglycerol acyltransferase	Reduce triglyceride (TG) levels, FDA (2004) [27,28,29]
**Icosapent ethyl (DB00159)**	VASCEPA^®^ (Amarin Pharma, Dublin, Ireland)	Fish	Small molecule (330.51 Da)	Reduces triglyceride synthesis by inhibiting 1,2-diacylglycerol acyltransferase	Reduces the risk of myocardial infarction, stroke, coronary revascularization, and unstable angina, FDA (2012) [30]
**Omega-3-carboxylic acids (DB09568)**	EPANOVA^®^ (AstraZeneca Pharmaceuticals, Landon, UK)	Fish	Small molecule (302.45 to 328.49 Da)	Reduces triglyceride synthesis by inhibiting 1,2-diacylglycerol acyltransferase	Reduce triglyceride (TG) levels, FDA (2014) [31]
**Fish oil triglycerides (DB13961)**	OMEGAVEN^®^ (Fresenius Kabi, Bad Homburg, Germany)	Fish	Small molecule (mixture of fatty acids, 700 to 1000 Da each)	Source of calories and essential fatty acids	PNAC, FDA (2018) [32]

^Ϯ^ Orphan drugs are commercially produced drugs for rare diseases. Because of their use to treat rare conditions, their production is not profitable and is often developed with government support.

**Table 2:** Components and their proportions in marketed fish oil products.

Drug Name	Component of Fish Oil and Its Form	Approximate Contents of Major Constituents	Drug Form and Usage
LOVAZA^®^ OMACOR^®^ OMTRYG^®^	Omega-3-acid ethyl esters	A 1 g capsule contains 465 mg of EPA and 375 mg of DHA	Liquid-filled gel capsule; used orally
VASCEPA^®^	Icosapent ethyl	0.5 g and 1 g of icosapent ethyl in 0.5 g and 1 g capsules, respectively	Amber colored, liquid-filled soft-gelatin capsule; used orally
EPANOVA^®^	Omega-3-carboxylic acids	850 mg of polyunsaturated fatty acids among which EPA and DHA are most abundant	Soft-gelatin capsule; used orally
OMEGAVEN^®^	Fish oil triglycerides	0.1 g of fish oil in 1 mL; EPA 13–26%, DHA 14–27%, and other fatty acids such as palmitic acid, oleic acid, palmitoleic acid, myristic acid, and arachidonic acid are present in low proportions	White-colored, homogenous emulsion; used intravenously

**Table 3:** Marine natural products being investigated in various stages of clinical trials.

Name	Company	Source Organism	Disease [NCT CODE]
**Phase III**			
Lurbinectedin (alkaloid)	Hoffmann-La Roche, Basel, Switzerland	*Ecteinascidia turbinata* (tunicate)	1. Study examining its combination with atezolizumab for higher-stage SCLC. [NCT05091567] 2. Study in combination of topotecan or irinotecan in patients with relapsed small-cell lung cancer. [NCT05153239]
Tetrodotoxin (alkaloid)	Wex Pharmaceuticals, Vancouver, Canada	*Pseudoalteromonas* sp., *Pseudomonas* sp., *Vibrio* sp. puffer fish (fugu).	Management of moderate or severe cancer-related pain. [NCT00726011]
Plitidepsin (depsipeptide)	PharmaMar, Madrid, Spain	*Aplidium albicans* (sea squirt)	Management of hospitalized patients with moderate COVID-19. [NCT04784559]
Marizomib	European Organization for Research and Treatment of Cancer-EORTC, Brussel, Belgium	*Salinispora tropica* (bacteria)	Non-small-cell lung cancer, pancreatic cancer, melanoma, lymphoma, multiple myeloma; newly diagnosed glioblastoma [NCT00667082, NCT03345095]
Plinabulin	Memorial Sloan Kettering Cancer Center; BeyondSpring Pharmaceuticals, New York City, NY, USA	Halimide (fungus)	Multiple myeloma; non-small-cell lung cancer, brain tumor [NCT05130827]
**Phase II**			
AGS-16C3F (MMAF)	Astellas Pharma Global Development, Tokyo, Japan	*Dolabella auricularia* (mollusk)	Metastatic renal cell carcinoma [NCT02639182]
Tisotumab vedotin (MMAE)	Seagen, Bothell, WA, USA	*Dolabella auricularia* (mollusk)	Cervical Cancer [NCT03438396]
Ladiratuzumab vedotin (MMAE)	Seagen, Bothell, USA	*Dolabella auricularia* (mollusk)	Small-cell lung cancer, non-small-cell lung cancer, squamous, non-small-cell lung cancer, non-squamous carcinoma, head and neck squamous cell carcinoma, esophageal squamous cell carcinoma, gastric adenocarcinoma, gastroesophageal junction adenocarcinoma, prostate cancer, melanoma [NCT04032704]
Telisotuzumab vedotin (MMAE)	AbbVie, North Chicago, IL, USA	*Dolabella auricularia* (mollusk)	Non-small-cell lung cancer [NCT03539536]
Enapotamab vedotin (MMAE)	Genmab, Copenhagen V, Denmark	*Dolabella auricularia* (mollusk)	Ovarian cancer, cervical cancer, endometrial cancer, non-small-cell lung cancer, thyroid cancer, melanoma, sarcoma [NCT02988817]
Disitamab vedotin (MMAE)	Seagen, Bothell, USA	*Dolabella auricularia* (mollusk)	Urothelial carcinoma, advanced cancer, gastric cancer, HER2-overexpressing gastric carcinoma, advanced breast cancer, solid tumors [NCT04879329]
CX-2029 (MMAE)	CytomX Therapeutics, South San Francisco, CA, USA	*Dolabella auricularia* (mollusk)	Solid tumors, head and neck cancer, non-small-cell lung cancer, diffuse large B-cell lymphoma, esophageal cancer [NCT03543813]
RC88 (MMAE)	RemeGen, Beijing, China	*Dolabella auricularia* (mollusk)	Solid tumors [NCT04175847]
W0101 (auristatin variant)	Pierre Fabre Medicament, Boulogne, France	*Dolabella auricularia* (mollusk)	Advanced or metastatic solid tumors, insulin-like growth factor type 1 receptor [NCT03316638]
ARX788 (auristatin variant)	Ambrx, La Jolla, CA, USA	*Dolabella auricularia* (mollusk)	HER2-positive metastatic breast cancer [NCT04829604]
XMT-1536	Mersana Therapeutics, Cambridge, MA, USA	*Dolabella auricularia* (mollusk)	Platinum-resistant ovarian cancer [NCT03319628, NCT04907968]
MORAb-202	Eisai, Bunkyo, Japan	*Halichondria okadai* (sponge)	Tumors [NCT04300556]
GTS-21	CoMentis, South San Francisco, CA, USA	*Nemertines* sp. (worm)	Alzheimer’s disease [NCT00414622]
Plocabulin (PM060184/PM184)	PharmaMar, Madrid, Spain	*Lithoplocamia lithiostoides* (sponge)	Solid tumors, advanced colorectal cancer [NCT03427268]
Soblidotin (auristatin PE; TZT-1027)	Daiichi Sankyo, Inc., Tokyo, Japan	Synthetic dolastatin 10 derivative	Non-small-cell lung cancer, sarcoma [NCT00061854, NCT00064220]
Synthadotin (Tasidotin; ILX-651)	Genzyme (Sanofi), Boston, MA, USA	Synthetic dolastatin 15 derivative	Melanoma, prostate cancer, non-small-cell lung carcinoma [NCT00068211, NCT00082134, NCT00078455]
Bryostatin 1	Neurotrope Bioscience, New York City, NY, USA	*Bugula neritina* (bryozoan)	Alzheimer’s disease, kidney cancer, acute myelogenous leukemia and myelodysplastic syndrome, colorectal cancer, myelodysplastic syndrome, relapsed multiple myeloma, Hodgkin disease, non-small-cell lung cancer, head and neck cancer, breast cancer, ovarian epithelial cancer, prostate cancer, cervical cancer, esophageal cancer, gastric cancer, relapsed acute myelogenous leukemia, esophageal cancer, gastric cancer. [NCT02221947, NCT00003968, NCT00136461, NCT00003171, NCT00002907, NCT00003936, NCT00005849, NCT00003443, NCT00003205, NCT00004008, NCT00005028, NCT00005965, NCT00005599, NCT00017342, NCT00006081]
**Phase I**			
CAB-ROR2-ADC (MMAE)	BioAtla, San Diego, CA, USA	*Dolabella auricularia* (mollusk)	Non-small-cell lung cancer, triple negative breast cancer, melanoma, head and neck cancer [NCT03504488]
FOR46 (MMAE)	Fortis Therapeutics, San Diego, CA, USA	*Dolabella auricularia* (mollusk)	Metastatic castration-resistant prostate cancer [NCT05011188]
ALT-P7 (MMAE)	Alteogen, Yuseong, S. Korea	*Dolabella auricularia* (mollusk)	HER2-positive breast cancer [NCT03281824]
MRG003 (MMAE)	Shanghai Miracogen, Shanghai, China	*Dolabella auricularia* (mollusk)	Advanced or metastatic gastric cancer [NCT05188209]
SGN-CD228A (MMAE)	Seagen, Bothell, USA	*Dolabella auricularia* (mollusk)	Cutaneous melanoma, pleural mesothelioma, HER2-negative breast neoplasms, non-small-cell lung cancer, colorectal cancer, pancreatic ductal adenocarcinoma [NCT04042480]
SGN-B6A (MMAE)	Seagen, Bothell, USA	*Dolabella auricularia* (mollusk)	Carcinoma, non-small-cell lung, squamous cell carcinoma of head and neck, HER2-negative breast neoplasms, esophageal squamous cell carcinoma, esophageal, adenocarcinoma, gastroesophageal junction adenocarcinoma, ovarian neoplasms, cutaneous squamous cell cancer, exocrine pancreatic adenocarcinoma, urinary bladder neoplasms, uterine cervical neoplasms, stomach neoplasms [NCT04389632]
Cofetuzumab pelidotin (auristatin variant)	AbbVie, North Chicago, USA	*Dolabella auricularia* (mollusk)	Cancer, non-small-cell lung cancer (NSCLC) [NCT04189614]
PF-06804103 (auristatin variant)	Pfizer, New York City, USA	*Dolabella auricularia* (mollusk)	Breast neoplasms [NCT03284723]
ZW-49 (auristatin variant)	Zymeworks, Vancouver, Canada	*Dolabella auricularia* (mollusk)	HER2-expressing cancers [NCT03821233]
A-166 (duostatin 5)	Sichuan Kelun Pharmaceutical Research Institute, Sichuan, China	*Dolabella auricularia* (mollusk)	Breast cancer [NCT05311397]
STI-6129 (duostatin 5)	Sorrento Therapeutics, San Diego, CA, USA	*Dolabella auricularia* (mollusk)	Light chain (AL) amyloidosis [NCT04316442]
Griffithsin	Center for Predictive Medicine, Louisville, KY, USA	Red alga	HIV prevention [NCT02875119]
Hemiasterlin (E7974)	Eisai, Bunkyo, Japan	Sponge	Cancer, malignant tumors [NCT00121732, NCT00130169, NCT00165802]

## Data Availability

All data generated or analyzed during this study are included in this published article.

## Supplementary Materials

The following supporting information can be downloaded at https://www.mdpi.com/1660-3397/20/8/528/s1: Figure S1. The flow diagram of the process used to select eligible studies; “n” represents the number of studies retrieved, and “N” represents the number of drugs retrieved. Table S1. Literature search strategy.
