# Correction: Haque et al. Marine Natural Products in Clinical Use. *Mar. Drugs* 2022, *20*, 528

**DOI:** 10.3390/md20100636

**Published:** 2022-10-13

**Authors:** Neshatul Haque, Sana Parveen, Tingting Tang, Jiaen Wei, Zunnan Huang

**Affiliations:** 1 Key Laboratory of Big Data Mining and Precision Drug Design of Guangdong Medical University, Key Laboratory of Computer-Aided Drug Design of Dongguan City, Key Laboratory for Research and Development of Natural Drugs of Guangdong Province, School of Pharmacy, Guangdong Medical University, No. 1 Xincheng Road, Dongguan 523808, China; 2 Centre for Cellular & Molecular Biology (CCMB), Hyderabad 500007, India; 3 Southern Marine Science and Engineering Guangdong Laboratory, Zhanjiang 524023, China

The authors would like to make corrections to a recently published paper [1].

## 1. Acknowledgment of the Web Resource

The authors wish to acknowledge the web resource at https://www.marinepharmacology.org/ (accessed on 30 September 2022) [2] as the source of the information presented in Table 3. We apologize for this unintentional omission.

## 2. Text Correction

In Table 3, the NCT codes listed below have been corrected.
**Names of Marine Natural Products****Wrong or Incomplete NCT Codes****Corrected NCT Codes****Phase III**Plitidepsin (depsipeptide)[NCT0478455][NCT04784559]Marizomib[NCT03345095][NCT00667082] [NCT03345095]**Phase II**RC88 (MMAE)[NCT0417584][NCT04175847]ARX788 (auristatin variant)[NCT03319628][NCT04829604]

The authors apologize for any inconvenience caused and state that the scientific conclusions are unaffected. The original publication has also been updated.
