# Supplementary material for: Marine Natural Products in Clinical Use
Source: Mar Drugs. 2022 Aug 18;20(8):528. doi: 10.3390/md20080528 (PMC9410185; doi:10.3390/md20080528)
Supplement: Supplementary file 1 [file marinedrugs-20-00528-s001.zip › Table S1.pdf]

Table S1. Literature search strategy

#1	((marine*) OR (sea)) OR (ocean)
#2	((((drug*) OR (product*)) OR (compound*)) OR (derivative*)) OR (agent*)
#3	(clinical*) OR (clinic*)
#4	FDA
#5	"1970/01/01" [Date - Publication]: "2022/06/30" [Date - Publication]
#6	#1 AND #2 AND #3 AND #4 AND #5
