# Supplementary figures and images for: Marine Natural Products in Clinical Use
Source: Mar Drugs. 2022 Aug 18;20(8):528. doi: 10.3390/md20080528 (PMC9410185; doi:10.3390/md20080528)

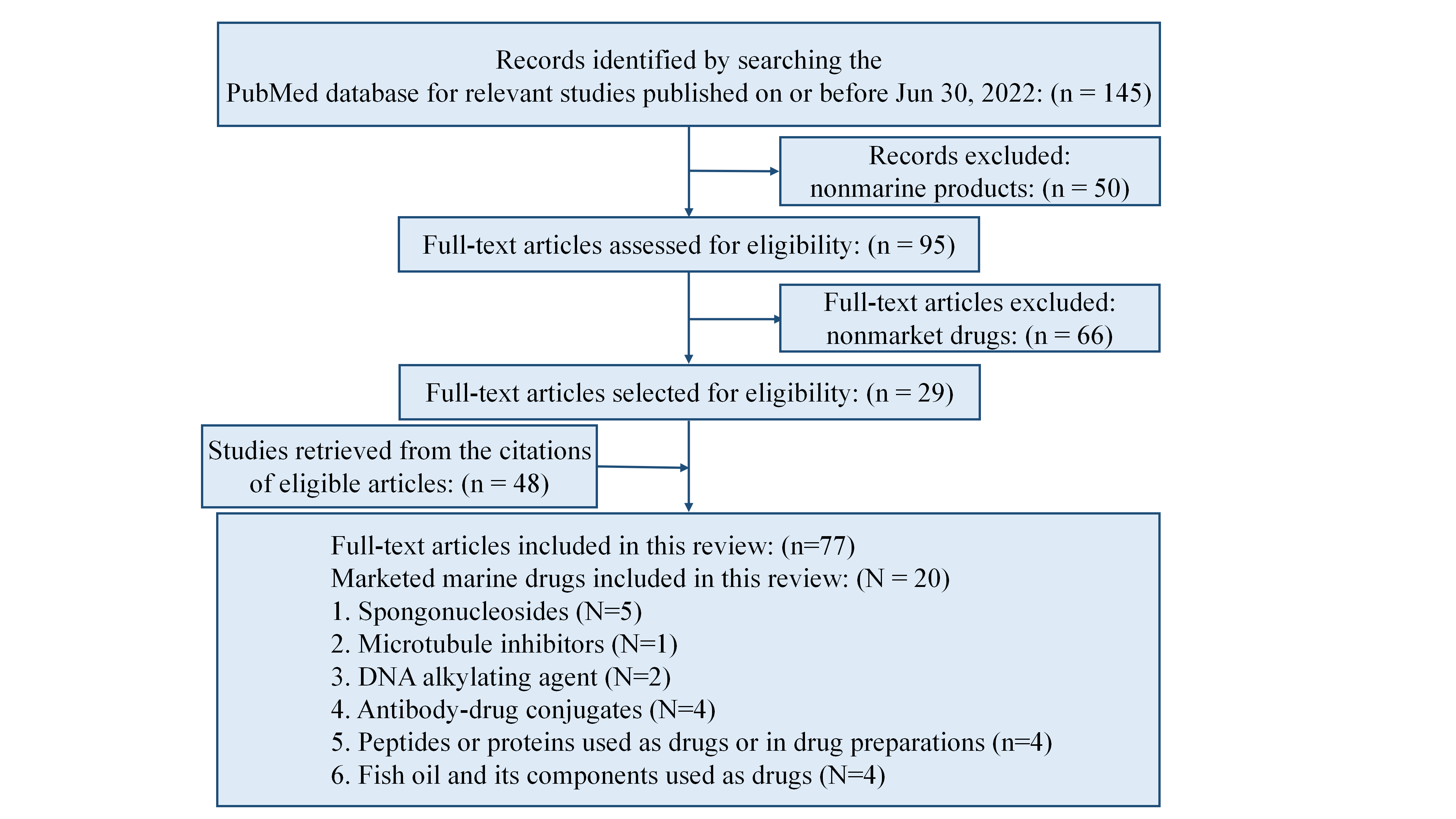

Supplement: Supplementary file 1 [file marinedrugs-20-00528-s001.zip › Figure S1.png]
